# Identification and characterization of protein interactions with the major Niemann–Pick type C disease protein in yeast reveals pathways of therapeutic potential

**DOI:** 10.1093/genetics/iyad129

**Published:** 2023-07-13

**Authors:** Natalie Hammond, Jamie Snider, Igor Stagljar, Kevin Mitchell, Kirill Lagutin, Matthew Jessulat, Mohan Babu, Paul H Teesdale-Spittle, Jeffrey P Sheridan, Stephen L Sturley, Andrew B Munkacsi

**Affiliations:** School of Biological Sciences, Victoria University of Wellington, Wellington 6012, New Zealand; Centre for Biodiscovery, Victoria University of Wellington, Wellington 6012, New Zealand; Donnelly Centre, University of Toronto, Toronto, Ontario M5S 3E1, Canada; Donnelly Centre, University of Toronto, Toronto, Ontario M5S 3E1, Canada; Department of Molecular Genetics, University of Toronto, Toronto, Ontario M5S 1A8, Canada; Department of Biochemistry, University of Toronto, Toronto, Ontario M5S 1A8, Canada; Mediterranean Institute for Life Sciences, Meštrovićevo Šetalište 45, HR-21000 Split, Croatia; Callaghan Innovation, Lower Hutt 5040, New Zealand; Callaghan Innovation, Lower Hutt 5040, New Zealand; Department of Biochemistry, Research and Innovation Centre, University of Regina, Regina, Saskatchewan S4S 0A2, Canada; Department of Biochemistry, Research and Innovation Centre, University of Regina, Regina, Saskatchewan S4S 0A2, Canada; School of Biological Sciences, Victoria University of Wellington, Wellington 6012, New Zealand; Centre for Biodiscovery, Victoria University of Wellington, Wellington 6012, New Zealand; School of Biological Sciences, Victoria University of Wellington, Wellington 6012, New Zealand; Centre for Biodiscovery, Victoria University of Wellington, Wellington 6012, New Zealand; Department of Biology, Barnard College-Columbia University, New York, NY 10027, USA; School of Biological Sciences, Victoria University of Wellington, Wellington 6012, New Zealand; Centre for Biodiscovery, Victoria University of Wellington, Wellington 6012, New Zealand

**Keywords:** rare disease, yeast model, lipid transport, lysosomal storage disease, neurodegenerative disease, sphingolipid, sterol

## Abstract

Niemann–Pick type C (NP-C) disease is a rare lysosomal storage disease caused by mutations in *NPC1* (95% cases) or *NPC2* (5% cases). These proteins function together in cholesterol egress from the lysosome, whereby upon mutation, cholesterol and other lipids accumulate causing major pathologies. However, it is not fully understood how cholesterol is transported from NPC1 residing at the lysosomal membrane to the endoplasmic reticulum (ER) and plasma membrane. The yeast ortholog of NPC1, Niemann–Pick type C–related protein-1 (Ncr1), functions similarly to NPC1; when transfected into a mammalian cell lacking NPC1, Ncr1 rescues the diagnostic hallmarks of cholesterol and sphingolipid accumulation. Here, we aimed to identify and characterize protein–protein interactions (PPIs) with the yeast Ncr1 protein. A genome-wide split-ubiquitin membrane yeast two-hybrid (MYTH) protein interaction screen identified 11 ER membrane-localized, full-length proteins interacting with Ncr1 at the lysosomal/vacuolar membrane. These highlight the importance of ER-vacuole membrane interface and include PPIs with the Cyb5/Cbr1 electron transfer system, the ceramide synthase complex, and the Sec61/Sbh1 protein translocation complex. These PPIs were not detected in a sterol auxotrophy condition and thus depend on normal sterol metabolism. To provide biological context for the Ncr1-Cyb5 PPI, a yeast strain lacking this PPI (via gene deletions) exhibited altered levels of sterols and sphingolipids including increased levels of glucosylceramide that mimic NP-C disease. Overall, the results herein provide new physical and genetic interaction models to further use the yeast model of NP-C disease to better understand human NP-C disease.

## Introduction

Niemann–Pick type C (NP-C) disease is a fatal neuro-visceral lysosomal storage disease without a cure or FDA-approved treatment (Vanier 2010). NP-C disease is caused by loss-of-function mutations in the genes that encode for NPC1, a cholesterol-binding protein residing in the membrane of the late endosome/lysosome (LE/Ly) (∼95% of cases) or NPC2, a soluble, cholesterol-binding protein in the LE/Ly lumen (∼5% of cases) (Carstea *et al.* 1997; Naureckiene *et al.* 2000; Vanier 2010). The current working model suggests low-density lipoprotein (LDL) containing cholesteryl ester, binds to the LDL-receptor at the plasma membrane (PM) where it is endocytosed and subsequently fused with the LE/Ly (Kwon *et al.* 2009). Here, lysosomal acid lipase acts to hydrolyze cholesteryl ester to release cholesterol. Next, it is theorized that NPC2 binds to cholesterol and transfers it to NPC1 at the membrane, functioning as a “tag-team” duo, where cholesterol is ultimately transferred out of the LE/Ly in an unknown manner.

Mutations in the genes encoding NPC1 or NPC2 result in an accumulation of various lipids including cholesterol (the disease “hallmark”), various sphingolipids including sphingomyelin, sphingosine, the gangliosides GM2 and GM3, glucosylceramide, and lactosylceramide, and the phospholipid bis(monoacylglycero)phosphate (Walkley and Vanier 2009; Lloyd-Evans and Platt 2010). As a result, a cascade of pathological events unfolds including neuronal dysfunction, autophagy, apoptosis, oxidative stress, inflammation, dysregulation of gene expression, and calcium homeostasis. Subsequently, this leads to hepatosplenomegaly in the viscera and numerous neurological defects including: hypotonia, loss of motor skills, supranuclear gaze palsy, ataxia, seizures, dysphagia, dysarthria, and dementia. Severe sufferers of NP-C disease succumb within 6 months of birth due to visceral organ failure. More commonly, patients gradually develop visceral disease with progressing neurological disease, ultimately causing death between 10 and 25 years of age (Vanier 2010; Patterson *et al.* 2017). Additionally, variation at the *NPC1* locus has been associated with several fundamental challenges in medicine including aging, neurodegeneration, obesity, and viral infections, suggesting that variation at the *NPC1* loci has widespread relevance beyond the rare NP-C disease (Erickson *et al.* 2008; Meyre *et al.* 2009; Tang *et al.* 2009; Carette *et al.* 2011).

Yeast is a unicellular eukaryotic organism, which is an effective model for studying many human diseases including neurodegenerative diseases (Botstein *et al.* 1997; Khurana and Lindquist 2010; Botstein and Fink 2011; Kachroo *et al.* 2022). This is largely thanks to the high degree of evolutionary conservation between the yeast and human genome, meaning fundamental aspects of cell biology and disease can be unraveled with unbiased genome-wide analyses. There are ∼50 human lysosomal storage disease-causing genes, of which 23 are conserved in yeast (Rajakumar *et al.* 2017). NP-C–related gene 1 (*NCR1*) encodes NP-C–related protein-1 (Ncr1), a yeast ortholog of human NPC1 with 35% amino acid sequence identity, a conserved structure, and is functionally interchangeable with mammalian NPC1 (Malathi *et al.* 2004). Similar to NPC1, Ncr1 can bind sterol via Npc2 (Winkler *et al.* 2019); however, Ncr1 is believed to have different functions in the yeast model relative to its function in sterol egress as observed by mammalian NPC proteins. This is supported by the observation that deletion of *NCR1* has no effect on sterol synthesis, esterification, or uptake (Malathi *et al.* 2004). Rather deletion of *NCR1* results in sphingolipid metabolism defects, whereby long-chain bases accumulate (Vilaça *et al.* 2014) and defects in the uptake/processing of lipid droplets at the vacuole (Tsuji *et al.* 2017; Winkler *et al.* 2019). NPC1 has been observed to physically interact with several sterol transfer proteins (STPs) present at membrane contact sites that bind cholesterol/ergosterol in their hydrophobic pockets (cavities) and facilitate transfer between 2 organelles in close proximity. The observation that NPC1 binds to STPs GramD1b and ORP5 was led by the initial identification of orthologous interactions with Ncr1 in yeast (Du *et al.* 2011; Höglinger *et al.* 2019).

Identification of the protein receiving cholesterol from mammalian NPC1 or yeast Ncr1 is not known and, once known, will increase our understanding of cholesterol transport as well as NP-C disease. Since the yeast model of NP-C disease has previously identified genetic and protein interactions conserved from yeast to mammals (Munkacsi *et al.* 2011; Colaco *et al.* 2020), we hypothesized the yeast model will also aid in detecting proteins that physically interact with NPC1/Ncr1.

## Materials and methods

### Yeast strains and plasmids

The split-ubiquitin membrane yeast two-hybrid (MYTH) L40 reporter strain (*MAT**a** trp1Δ his3Δ leu2Δ ade2Δ LYS2::lexA-HIS3 URA3::lexA-lacZ GAL4*) and L2, L3, and pPR3N MYTH plasmids were used for protein–protein interaction (PPI) analyses (Snider *et al.* 2010). Wild-type (WT) strains BY4741 (*MAT**a** his3Δ1 leu2Δ0 met15Δ0 ura3Δ0*), Y7092 (*MAT**α** can1Δ::STE2pr-Sp_HIS5; lyp1Δ; his3Δ1 leu2Δ0 ura3Δ0 met15Δ0 LYS2*), and representative strains from the *kanMX* gene deletion and decreased abundance by mRNA perturbation (DAmP) libraries in the BY4741 background (Open Biosystems) were used for genetic analyses. Single deletion of *NCR1* in Y7092 was constructed via PCR-mediated disruption (natMX) as previously described (Munkacsi *et al.* 2011). Double gene mutants (*ncr1ΔxxxΔ or ncr1Δxxx-DAmP*) were constructed by synthetic genetic array analysis (Tong *et al.* 2001). The strain used for colocalization analysis was constructed herein (Mat**a**  *NCR1-*mScarlet-I, *CYB5*-GFP::*His5*, *his3Δ1 leu2Δ0 met15Δ0*  *ura3Δ0*).

### MYTH analysis

MYTH was conducted as previously described (Snider *et al.* 2010, 2013) where plasmids expressing prey proteins (NubG-Xxx) were transformed into an Xxx-Cub-TF tagged yeast strain. Transformed cells were plated on appropriate agar for plasmid selection [SD-tryptophan (SD-W)] and incubated at 30°C for 2–4 days. To test for an interaction between bait and prey proteins, prey plasmids were transformed into bait strains where resulting transformants were grown in 2 mL of SD-W broth overnight at 30°C with agitation, serial diluted (1:5) in a 96-well plate (Jet BioFil), and pinned onto various agar media to measure the strength of the interaction: control (SD-W), low stringency agar [SD-W histidine (SD-WH)], and high stringency agar [SD-WH + 5-bromo-4-chloro-3-indolyl-β-D-galactopyranoside (X-Gal)]. Plates were incubated for 3 days at 30°C and imaged with a digital camera (Canon EOS 600D).

### MYTH bait construction

The MYTH bait strain was constructed as previously described (Snider *et al.* 2010, 2013). Primers were designed specific to *NCR1*: the forward primer consisted of a 45 bp sequence at the C-terminal excluding the stop codon, paired with a 20 bp sequence from the L2 (or L3) plasmid; the reverse primer consisted of a 45 bp sequence after the ORF, paired with a 20 bp sequence from the L2 (or L3) plasmid (Supplementary Table 1). The tagging cassette was amplified by PCR, transformed into the MYTH reporter strain, plated on YPD + G418 agar, and incubated at 30°C for 3 days. Correct tagging of *NCR1* was verified with Sanger sequencing of a PCR product amplified using a forward primer (25 bp sequence localized 140 bp upstream from the *NCR1* stop codon) along with a reverse primer sequence specific to the kanMX marker (Supplementary Table 1).

### Localization analysis of Ncr1-Cub-YFP-TF

Localization of Ncr1-Cub-TF was investigated via Yellow Fluorescent Protein (YFP) fusion as previously described (Snider *et al.* 2010). The Ncr1-Cub-YFP-TF strain, constructed using the L3 plasmid, was grown in selection broth (YPD + G418) overnight at 30°C with agitation, diluted to a starting cell density of OD_660_ = 0.2 in 3 mL of YPD, incubated at 30°C with agitation for 5 h, centrifuged, washed with ddH_2_O, and resuspended in 30 µL ddH_2_O. For imaging, 1 µL of cells was mounted onto a microscope slide and imaged using an IN Cell Analyzer 6500 HS (General Electric) confocal microscope with a 60 × objective lens. Differential interference contrast (DIC) was used to capture cell shape and vacuole; YFP was detected using the green filter excited at 488 nm and emitted at 524 nm with 1 s exposure. Images were prepared using FIJI ImageJ.

### NubG/I test

To confirm newly constructed bait strains did not self-activate and interact with nonspecific prey proteins, a NubG/I test using prey proteins NubI (positive control) and NubG (negative control) was performed (Snider *et al.* 2010). NubI and NubG plasmids were transformed into Ncr1-Cub-TF, and resulting transformants were grown in 2 mL SD-W broth overnight at 30°C with agitation. Saturated cultures were serial diluted (1:5) into a 96-well plate, pinned onto control (SD-W), low stringency agar (SD-WH), and high stringency agar (SD-WH + X-Gal) agar, incubated for 3 days at 30°C and imaged with a digital camera (Canon EOS 600D).

### Full-length prey construction

Full-length prey constructs were generated using *in vivo* homologous recombination as previously described (Ma *et al.* 1987). First, prey genes (*CYB5*, *LIP1*, *MHF1*, *PGA3*, *PHS1*, *SNF1*, *SSS1*, *VBA1*, *VMA3*, *VMA9*, *VMA11*, *VOA1*, and *YSY6*) were amplified from BY4741-derived genomic DNA using primers with a 20–25 bp sequence homologous to the beginning and end of the gene of interest, paired with a 40 bp sequence homologous to pPR3N (Supplementary Table 1). pPR3N was digested with SfiI (Thermo Scientific) at 50°C for 6 h. Next, PCR product (50 µL) and linearized plasmid (5 µL) were cotransformed into a MYTH reporter strain (L40) with 40 min of heat shock at 42°C, followed by outgrowth in YPD broth for 3 h prior to plating on SD-W agar with subsequent incubation at 30°C for 3 days. Single colonies were then grown in 3 mL SD-W overnight at 30°C with agitation. New full-length prey plasmid was extracted from the saturated yeast culture using the NucleoSpin Plasmid DNA purification (Macherey-Nagel) with the addition of 150 µL of 0.5 mm glass beads (DNature) to the first buffer and vigorously vortexed. To increase plasmid copy number, a bacterial transformation was performed using 15 µL of plasmid extracted from yeast, followed by subsequent plasmid DNA purification from *Escherichia coli*. Retransformed plasmid was confirmed for correct tagging with Sanger sequencing.

### Co-immunoprecipitation analysis

PPIs identified using MYTH were independently validated using co-immunoprecipitation (Co-IP) as previously described (Babu *et al.* 2012). Tandem affinity protein (TAP)-tagged Ncr1 and wild-type strains were transformed with the hemagglutinin (HA)-tagged prey plasmids (Cyb5-HA, Lip1-HA, Pga3-HA, Phs1-HA, Sss1-HA, and Ysy6-HA) using the lithium acetate/single stranded carrier DNA/PEG method (Gietz and Schiestl 2007). Resulting transformants were grown overnight in SD-Ura and washed before resuspension in nonrepressing YPGE media for 6 h. Plasmid expression was induced for 1 h with the addition of 2% galactose. Cells were pelleted at 3000 rpm for 4 min and resuspended in 500 µL IPLB calmodulin buffer (50 mM KOAc, 2 mM Mg(Ac)_2_, 2 mM CaCl_2_, 20 mM HEPES pH 7.4) with 1% Triton and 1 × protease inhibitor cocktail (EMD Millipore). An equal volume of glass beads was added, and cells were lysed upon vortexing at maximum speed for 5 min. Heavy debris and unbroken cells were pelleted by centrifugation at 3000 rpm for 4 min and remaining lysate transferred to a new tube. Then, 50 µL calmodulin sepharose 4B beads (GE Healthcare) was added to the lysate, incubated overnight on a rotator, and centrifuged for 2 min at 2000 rpm. Next beads were washed once with 800 µL ice cold IPLB calmodulin buffer with 0.1% Triton, followed by 5 washes with calmodulin wash buffer (150 mM NaCl, 1 mM Imidazole, 2 mM CaCl_2_, 30 mM Tris pH 7.9), with aspiration and centrifugation after each wash. Samples were resuspended in 50 µL SDS loading buffer, heated at 95°C for 5 min, loaded in a 10% acrylamide gel, and processed in a western blot analysis. Primary mouse monoclonal anti-HA tag (Santa Cruz sc-7392) and secondary goat antimouse HRP conjugated (Santa Cruz sc-2005) were used as the antibodies, and results were visualized with a Kodak 4000 mm Imaging Station.

### Agar-based bioactivity

Viability of cells was determined as previously described (Munkacsi *et al.* 2011). Synthetic Complete/Synthetic Dropout (SC/SD) solid agar was prepared with an appropriate concentration of the drug of interest. Cells were grown overnight in 2 mL of selection media at 30°C with agitation, diluted 1:5 across 6 wells of a 96-well plate (Jet BioFil), plated onto drug-supplemented plates using a 96-prong pinning tool, incubated at 30°C for 3 days, and imaged with a digital camera (Canon EOS 600D).

### Anaerobiosis (sterol uptake) assay

Sterol uptake was investigated via anaerobic growth analysis as previously described (Munkacsi *et al.* 2011). Cells were incubated in 2 mL selection media overnight at 30°C with agitation, diluted 1:5 across 6 wells of a 96-well plate (Jet BioFil), and plated on SC/SD agar supplemented with 20 µg/mL ergosterol (Sigma-Aldrich) and 0.5% Tween 80 (Sigma-Aldrich) that was incubated in an anaerobic chamber system (BD GasPakTM) with activated charcoal and 3 carbon sachets (BD GasPakTM) or aerobic under standard oxygen conditions. Plates were incubated for 3–5 days (depending on the strain) in the dark at 30°C.

### Sphingolipid extraction

Sphingolipids were extracted as previously described (Kondo *et al.* 2014). Cells were grown overnight in 2 mL of selection media at 30°C with agitation. The overnight culture was then diluted to a starting cell density of OD_660_ = 0.25 with SC media and incubated at 30°C with agitation (24 h, postdiauxic phase). Cell density was converted to cell count and 9.25 × 10^7^ cells were harvested, washed with ice cold ddH_2_O, treated with 5% trichloroacetic acid (Sigma-Aldrich) at 4°C for 20 min, centrifuged at 13,000 rpm for 5 min, and stored at −80°C for at least 24 h prior to extraction. For lipid extraction, 1 mL of extraction solvent [ethanol (Pure Science): ddH_2_0: diethylether (Thermo Fisher): pyridine (Thermo Fisher): ammonium hydroxide (Sigma-Aldrich) (15:15:5:1:0.018)] was added to the cell pellets and vortexed for 2 min. Samples were incubated in a water bath at 60°C for 15 min, followed by centrifugation at 13,000 rpm for 10 min where supernatant was collected and transferred to a new tube. Samples underwent a second extraction whereby 1 mL of extraction solvent was again added to the sample tube, vortexed, incubated at 60°C, and centrifuged, and organic phases from the 2 rounds were combined. Lipid extracts were dried at room temperature overnight under a stream of nitrogen using a sample concentrator (Techne) and stored at −80°C.

### Ultra-high performance liquid-chromatography mass spectrometry analysis of sphingolipids

A wide range of polarity lipid compounds were quantified using an in-house ultra-high performance liquid-chromatography mass spectrometry (UHPLC-MS) method based on a previously described method (Isaac *et al.* 2011). Dry samples were resuspended in 200 µL with chloroform/methanol/water (4:4:1.25). Analysis was carried out using a Waters H-class UPLC chromatography system interfaced to a Waters G2XS QTof mass spectrometer with electrospray ionization source. Chromatography was carried out using a Waters Premier CHS C18 1.7-micron 2.1 mm × 150 mm column. Elution solvents were (1) acetonitrile/water (3:2) with 10 mM ammonium formate and 0.1% formic acid and (2) 2-propanol/acetonitrile (9:1) with 10 mM ammonium formate and 0.1% formic acid added. A gradient profile was employed, and initial conditions were 32% B, held at this composition for 1.5 min following sample injection, increased linearly to 45% B at 4 min from injection, 52% B at 5 min, 58% B at 8 min, 67% B at 11 min, 70% B at 14 min, 75% B at 18 min, 97% B at 21 min, held at 97% B until 25 min, then returned to initial conditions at 25.5 min. The column was allowed to equilibrate for 4.5 min before a subsequent injection was made. A flow rate of 0.2 mL/min was employed, a column temperature of 55°C, and injection volume of 1 µL. Mass spectroscopy data was collected in both positive and negative ionization modes, each mode requiring a separate chromatography run. Date was collected in MSE mode, i.e. both low collision energy at 2 V and high collision energy using a ramp from 30 to 50 V to provide fragmentation data. Instrument control was achieved using Waters MassLynx V4.2 and post data processing using Nonlinear Dynamics Progenesis QI software. Identification of individual lipids was achieved by importing the LIPID MAPS database of compounds into Progenesis as an SDF database and then screening ions detected based on both accurate mass and fragmentation (Sud *et al.* 2007). Candidate identifications were then manually screened based on expected retention time ranges for the classes of compounds observed and known occurrence of lipid classes in yeast, an accurate mass within 5 ppm, and observation of one or more predicted fragments in the high fragmentation energy channel. Statistical analysis of the quantification data was performed using a Brown–Forsythe and Welch ANOVA test and corrected for using a Games–Howell's multiple comparisons test. Adjusted *P*-values are reported (GraphPad Prism version 8.0).

### Neutral lipid extraction

A modified Matyash extraction method was used to extract neutral lipids (Matyash *et al.* 2008). Cells were grown overnight in 2 mL of selection media at 30°C with agitation. The overnight culture was then diluted to a starting cell density of OD_660_ = 0.25 with SC media and incubated at 30°C with agitation until reaching OD_660_ = 1 (6–9 h, early-log phase). Cell density was converted to cell count and 1.85 × 10^8^ cells were harvested, centrifuged, washed with ddH_2_O, and stored at −80°C for at least 24 h before extraction. To extract lipids, 150 µL of 0.5 mm glass beads (DNature) and 400 µL isopropanol were added to the cell pellet that was briefly vortexed and incubated overnight at 4°C while shaking at 1000 rpm (ThermoMix, Eppendorf). To evaporate isopropanol, cell pellets were dried in a speed vac for 30 min, followed by the addition of 700 µL of methyl tertiary-butyl ether (MTBE)/methanol (MeOH) (10:3, v/v). Samples were incubated at 4°C while shaking at 1000 rpm (ThermoMix, Eppendorf). After 1 h, 200 µL of water was added to the extract to aid in phase separation and incubated for a further 20 min before phase separation via centrifugation at 13,000 rpm for 10 min. The lipid-containing upper phase was collected into a new tube, and extracts were dried at room temperature using a nitrogen sample concentrator (Techne) and stored at −80°C.

### Supercritical fluid chromatography-mass spectrometry analysis of neutral lipids

Neutral lipids were measured using an in-house adaptation of supercritical fluid chromatography-mass spectrometry as previously described (Broughton *et al.* 2020). Analysis was carried out on dried lipid extracts resuspended in 200 µL of chloroform/MeOH (2:1) via a Waters UPC2 analytical supercritical chromatography system interfaced to a Waters G2XS Q-ToF mass spectrometer with atmospheric pressure chemical ionization source, using a Waters Premier CHS C18 1.7-micron 2.1 mm × 150 mm column. Elution solvents were (1) CO_2_ and (2) methanol/ethanol (1:1 v/v) with 20 mM ammonium formate, 0.1% v/v formic acid, and 2% water v/v. Methanol (0.1% concentrated ammonia solution v/v) was used as the post column isocratic flow post column prior to the flow splitter providing flow to both the mass-spectrometer and back pressure regulator, with a flow rate of 0.3 mL/min. A gradient profile was employed, initial conditions were 1% B, held at this composition for 1 min following sample injection, increased linearly to 2.5% B at 6 min from injection, 5% B at 8 min, 20% B at 13 min, 30% B at 15 min, 35% B at 16 min, 40% B at 19 min, held at 40% B until 25 min, reduced to 20% B at 26 min, then returned to initial conditions at 26.5 min. The column was allowed to equilibrate for 4.5 min before a subsequent injection was made. A flow rate of 0.8 mL/min was used for eluting solvents until 16 min and then linearly reduced to 0.6 mL/min at 19 min, this flow rate was maintained until 26 min when it was linearly increased to 0.8 mL/min at 26.5 min, then maintained at this rate until the end of the run. The back pressure regulator was set to 1500 psi and column temperature of 60°C. An injection volume of 1 µL was used for all samples. Identification of individual lipids was achieved by screening ions based on both accurate mass and fragmentation against the LIPID MAPS database (Sud *et al.* 2007), using the same methodology as described above for UHPLC-MS data analysis. Candidate identifications were then manually screened based on expected retention time ranges for the classes of compounds observed and known occurrence of lipid classes in yeast, an accurate mass within 5 ppm, and observation of one or more predicted fragments in the high fragmentation energy channel. Statistical analysis of the quantification data was performed using a Brown–Forsythe and Welch ANOVA test and corrected for using a Games–Howell's multiple comparisons test. Adjusted *P*-values are reported (GraphPad Prism version 8.0).

### Growth analysis

Liquid growth assays were conducted in 96-well plates (Jet Biofil) as previously described (Coorey *et al.* 2014). Cells were incubated in 2 mL of selection media and grown overnight at 30°C with agitation. Then 95 µL of SC media and 5 µL of 0.128 × 10^7^ cells/mL were added to each well. The plate was shaken at 1000 rpm for 60 s (MixMate Eppendorf), before absorbance was measured at 590 nm every 60 min for 48 h using an Envision 2102 Multilabel plate reader (Perkin Elmer). Growth (OD_590_) over time (h) was calculated via mean ± SEM for triplicate samples. An unpaired multiple *t*-test analysis was performed using GraphPad Prism version 8.0.

### Colocalization analysis

The Ncr1-mScarlet-I Cyb5-GFP strain was grown overnight at 30°C with agitation, diluted to a starting cell density of 0.255 × 10^7^ cells/mL in 2 mL of SC, incubated at 30°C with agitation for 5 h, centrifuged, washed with PBS, and resuspended in 50 µL PBS. For imaging, 1 µL of cells was mounted onto a microscope slide and imaged using an IN Cell Analyzer 6500 HS (General Electric) confocal microscope with a 60 × objective lens. DIC was used to capture cell shape and vacuole, GFP was detected using the green filter excited at 488 nm and emitted at 524 nm with a 2 s exposure, and the mScarlet-I was detected using the orange filter excited at 561 nm and emitted at 605 nm with a 3 s exposure. Images were prepared using FIJI ImageJ.

### Mapping the interaction site between bait and prey proteins

Alanine mutagenesis was used to determine the contribution of specific residues to a PPI as previously described (Cunningham and Wells 1989), specifically via a combinatorial approach whereby a cluster of 6 amino acids was mutated (Lefèvre *et al.* 1997). Topology of prey proteins was predicted by Protter (Omasits *et al.* 2014) to help determine sites of mutagenesis; only amino acids predicted to protrude into the cytosol were targeted for mutagenesis. After codon optimization, mutant genes and cloned sequences were incorporated into the pPR3N vector by Twist Bioscience. Codon-optimized sequences for each gene mutant and the corresponding wild-type (nonmutated) sequence are listed in Supplementary Table 2. Plasmids were received from Twist Bioscience and extracted using a NucleoSpin Plasmid DNA purification kit (Macherey-Nagel). MYTH analysis was performed (as described above) to determine if residues were required for prey interaction with Ncr1-Cub-TF.

## Results

### MYTH analysis identifies 6 PPIs with Ncr1

To identify proteins interacting with Ncr1, a split-ubiquitin MYTH assay was performed (Supplementary Fig. 1); this method screens for PPIs via the fusion of a bait-expressed C-terminus of ubiquitin (Cub) and a prey-expressed N-terminus of ubiquitin that upon bait–prey interaction forms a functional ubiquitin molecule that activates ubiquitin-specific proteases and associated reporter genes (Stagljar *et al.* 1998; Scheper *et al.* 2003; Dietschy *et al.* 2007; Paumi *et al.* 2008, 2009; Rizzolo *et al.* 2017). Although several proteome-wide analyses have been done to identify PPIs with Ncr1 in yeast using affinity capture-RNA, dihydrofolate reductase and TAP-tag methodologies (Krogan *et al.* 2006; Tarassov *et al.* 2008; Babu *et al.* 2012), only 3 PPIs with Ncr1 (Npc2, Pmc1, and Pdr5) have been identified using 2 independent assays, and 2 of these (Npc2 and Pmc1) have been further investigated in higher eukaryotic models (Snider *et al.* 2013; Winkler *et al.* 2019; Colaco *et al.* 2020).

The Ncr1-Cub-TF bait strain was constructed and validated in the L40 MYTH starting strain (Supplementary Fig. 2) prior to PPI analysis. The NubG/I test is a genetic assay performed to ensure the bait construct is properly inserted into the membrane with the C-terminal tag in the cytosol and to ensure the bait is not self-activating—the bait does not itself activate the reporter genes. Mis-targeting of the bait protein may lead to instability and degradation of the bait protein ultimately leading to self-activation (Fetchko and Stagljar 2004; Paumi *et al.* 2007; Snider *et al.* 2010; Petschnigg *et al.* 2012). NubI-Ost1 contains a nonmutated N-terminal of ubiquitin where regardless of bait–prey interaction, ubiquitin will spontaneously reconstitute and result in growth on selection agar. NubG-Ost1 contains a mutated N-terminal of ubiquitin (isoleucine 13 to glycine mutation) and does not spontaneously form reconstituted ubiquitin unless bait and prey proteins interact. Ncr1-Cub-TF transformed with NubI-Ost1 resulted in cell growth on all selection agar plates (control, low stringency and high stringency). Ncr1-Cub-TF transformed with NubG-Ost1 resulted in cell growth on the control plate alone, did not self-activate and therefore was deemed suitable for further investigation (Supplementary Fig. 2a). To ensure tagging of Ncr1 did not disrupt protein localization, *NCR1* was tagged with Cub-YFP-TF via PCR amplification using the YFP-containing L3 plasmid. Fluorescent microscopy of Ncr1-Cub-YFP-TF depicted fluorescence localized to the vacuolar membrane (Supplementary Fig. 2b), which was an expected result given Ncr1 is localized to the vacuolar membrane (Zhang *et al.* 2004).

**Fig. 1.**
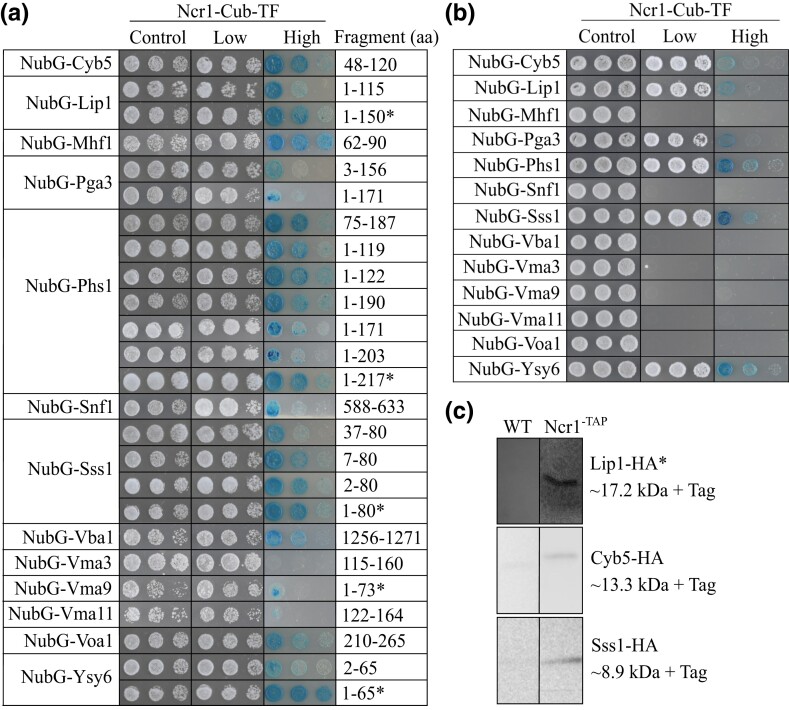
PPIs with Ncr1 using MYTH and co-IP analysis. MYTH was conducted where plasmids expressing prey proteins (NubG-Xxx) were transformed into an Ncr1-Cub-TF bait strain. Serial dilutions (1:5) of saturated transformants were plated on selection stringency agar [control (SD-W), low (SD-WH), high (SD-WH + X-Gal)] and incubated at 30°C for 3 days. Shown are cell dilutions 1:5, 1:25, and 1:125. a) Prey plasmids recovered from the prey plasmid library were retransformed into MYTH bait Ncr1-Cub-TF. b) Full-length prey proteins were constructed and transformed into Ncr1-Cub-TF. c) Co-IP analysis detected physical interactions between Cyb5-HA, Lip1-HA, and Sss1-HA with TAP-tagged Ncr1. Cyb5-HA, Lip1-HA, and Sss1-HA plasmids were transformed into WT and Ncr1-TAP tagged strains; co-IP analysis and subsequent western blot probed with anti-HA in WT and Ncr1-TAP tagged strains. *Digitonin was used instead of triton for the detergent. Expected sizes recovered on the western blot are indicated.

Screening Ncr1-Cub-TF against cDNA and gDNA prey libraries made up of fragments and full-length proteins (NubG-Xxx) recovered 13 interactors including: Cyb5, Lip1, Mhf1, Pga3, Phs1, Snf1, Sss1, Vba1, Vma3, Vma9, Vma11, Voa1, and Ysy6 (Fig. 1a). Cytochrome b5 (Cyb5) is a membrane-bound hemoprotein that functions as an electron carrier for several membrane-bound oxygenases and is localized to the ER; Lip1 (Lag1/Lac1 interacting protein) is a component of the ceramide synthase complex required for synthesis of ceramides and is localized to the ER; Mhf1 (Mph1-associated histone-fold protein) is a component of the kinetochore functioning in meiosis and mitosis and localizes to the nucleus; Pga3 (processing of Gas1p and ALP) is a NADH-dependent cytochrome b5 reductase functioning at the ER and PM; Phs1 (PTPLA homolog involved in sphingolipid biosynthesis) catalyzes the third reaction of the long-chain fatty acid elongation cycle and localizes to the ER; Snf1 (sucrose nonfermenting) is a catalytic subunit of the AMP-activated protein kinase complex localized to the cytoplasm and nucleus; Sss1 (Sec61 suppressor) is a member of the Sec61 complex that mediates protein translocation across the ER; Vba1 (vacuolar basic amino acid transporter) is an amino acid transporter required for vacuolar uptake of histidine and lysine localized to the vacuole; Vma3, Vma9, and Vma11 (vacuolar membrane ATPase) are members of the proton-conducting pore V0 complex of the vacuolar (H+)-ATPase (V-ATPase) localized to the vacuole; Voa1 (V0 assembly protein) is an accessory protein of the V0 complex of V-ATPase localized to the vacuole and ER; and Ysy6 (suppressor of SecY) is thought to interact with target proteins during translocation into the lumen of the ER (Cherry *et al.* 2012; Bateman *et al.* 2021).

Cell growth on control (SD-W) agar ensured the prey plasmid was successfully transformed into Ncr1-Cub-TF. Growth on low stringency agar (SD-WH) tested the activation of *HIS3*; activation of the *HIS3* reporter gene upon PPI makes transformants prototrophs for *HIS3* and thus can survive on media lacking histidine. Growth on high stringency agar (SD-WH + X-Gal) tested the activation of *LacZ*. Expression of *LacZ* produces β-galactosidase which turns colonies blue in color when plated on agar containing X-Gal. Growth on these various selection media reflect strength/stringency of the interaction (Fig. 1a). Growth on control and low stringency agar appeared consistent with visible growth across all cell dilutions. Growth on high stringency agar showed variable intensities of blue colony growth, suggesting the PPIs occur at varying strengths: “strong” interactions, where all 3 spot dilutions displayed cell growth on high stringency agar included NubG-Mhf1, NubG-Phs1, and NubG-Ysy6; “medium” interactions, where 2 spot dilutions displayed cell growth on high stringency agar included NubG-Cyb5, NubG-Lip1, NubG-Sss1, NubG-Vba1, and NubG-Voa1 and “weak” interactions, where one or no spot dilution displayed cell growth on high stringency agar included NubG-Pga3, NubG-Snf1, NubG-Vma3, NubG-Vma9, and NubG-Vma11.

Because 8 of 13 interactors of Ncr1-Cub-TF appeared only as fragments, genes encoding full-length prey proteins were amplified by PCR using gene-specific primers with a flanking sequence to the prey plasmid pPR3N and cotransformed with linearized prey plasmid cassette into a MYTH reporter strain (L40); a summary of this process is shown in (Supplementary Fig. 3). Newly constructed plasmids (NubG-Xxx) were transformed into Ncr1-Cub-TF, and PPIs were determined by growth on MYTH selection agar. Positive PPIs with Ncr1 were detected for 6 of these 13 full-length proteins (Fig. 1b). All interactions appeared to show equivalent cell growth, suggesting all transformations with Ncr1-Cub-TF were successful and cell density across samples comparable. With low stringency agar, cells grew well for 6 interactors (NubG-Cyb5, NubG-Lip1, NubG-Pga3, NubG-Phs1, NubG-Sss1, and NubG-Ysy6). The remaining 7 interactors (NubG-Mhf1, NubG-Snf1, NubG-Vba1, NubG-Vma3, NubG-Vma9, NubG-Vma11, and NubG-Voa1) showed no growth, suggesting these full-length proteins did not interact with Ncr1-Cub-TF. Growth on high stringency agar showed variable intensities of blue colony growth, suggesting the PPIs occur at varying strengths. NubG-Cyb5, NubG-Lip1, and NubG-Pga3 showed blue colony growth in the first cell dilution, while NubG-Phs1, NubG-Sss1, and NubG-Ysy6 showed a stronger interaction with cell growth visible in 2 cell dilutions. Overall, these results indicate that Ncr1 physically interacts with 6 proteins (Cyb5, Lip1, Pga3, Phs1, Sss1, and Ysy6).

### Co-IP detects a PPI between Ncr1-Cyb5, Ncr1-Lip1, and Ncr1-Sss1

MYTH screening is a powerful first step in identifying interactors of Ncr1, whereby identified prey interactors must be further validated by independent PPI assays. To validate Ncr1 interactors detected via MYTH, a second independent PPI assay was undertaken on the 6 full-length proteins identified to interact with Ncr1 via MYTH. Here, co-IP analysis was conducted for TAP-tagged Ncr1 or WT transformed with HA-tagged prey plasmids (Xxx-HA). Western blot analysis detected PPIs for Ncr1 with Cyb5-HA, Lip1-HA, and Sss1-HA; these PPIs exhibited bands in only the Ncr1-TAP strain (Fig. 1c). The PPI with Lip1-HA was recovered using digitonin as the detergent, while the PPIs with Sss1-HA and Cyb5-HA were recovered using Triton as the detergent. Therefore, this co-IP analysis validated 3 of the 6 interactions identified via MYTH.

### Ncr1 interacts with Sec61 and other proteins in the Sec61 protein translocation complex

**Fig. 2.**
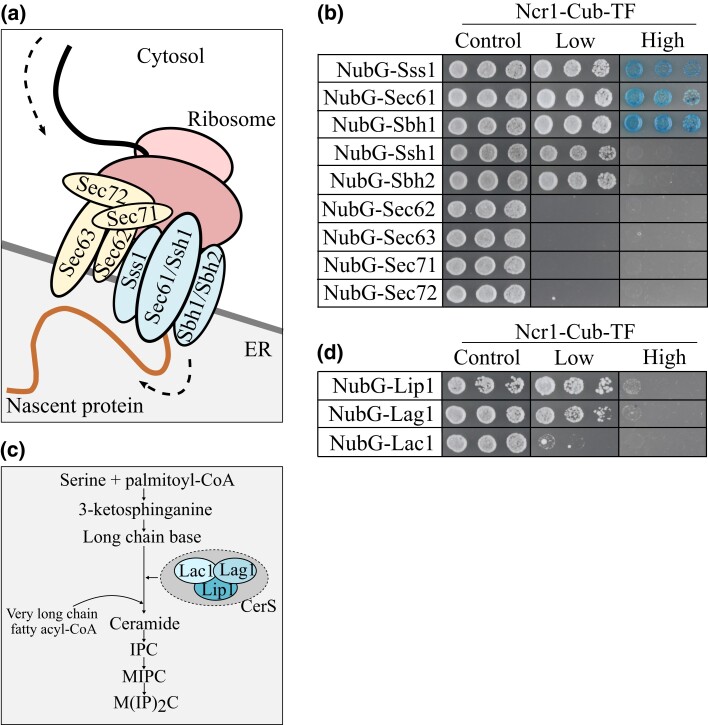
Members of the Sec61 protein translocation complex and the ceramide synthase complex physically interact with Ncr1. a) Simplified cartoon showing Sss1 functioning as part of the Sec61 protein translocation complex (Sss1, Sec61/Ssh1, Sbh1/Sbh2). Also functioning in protein translocation is the Sec63 complex (Sec62, Sec63, Sec71, and Sec72). Ribosome processing the mRNA binds to the Sec protein complexes on the ER membrane. Newly translated protein is transported into the ER through these complexes for further processing. Topology based on Itskanov and Park (2019). b) Full-length prey protein plasmids (NubG-Sss1, NubG-Sec61, NubG-Sbh1, NubG-Ssh1, NubG-Sbh2, NubG-Sec62, NubG-Sec63, NubG-Sec71, and NubG-Sec72) were transformed into Ncr1-Cub-TF. c) Simplified cartoon showing Lip1 functioning as part of the ceramide synthase complex (Lag1, Lac1, and Lip1) which converts sphingoid bases to ceramide in sphingolipid biosynthesis. d) Prey protein plasmids (NubG-Lip1, NubG-Lag1, and NubG-Lac1) were transformed into Ncr1-Cub-TF. For b) and d), serial dilutions (1:5) of saturated transformants were plated on selection stringency agar [control (SD-W), low (SD-WH), high (SD-WH + X-Gal)] and incubated at 30°C for 3 days. Shown are cell dilutions 1:5, 1:25, and 1:125.

Of the 6 full-length proteins that interacted with Ncr1-Cub-TF, 2 of these function in different protein complexes (Sss1 and Lip1). It was reasoned that if the protein interactions with Ncr1 are true, then it is likely that Ncr1 will also interact with the other proteins that make up these complexes. Plasmids for the expression of proteins present in these protein complexes were constructed and analyzed for PPIs via MYTH. Sss1 is part of the Sec61 protein translocation complex (Sec61, Sbh1, and Sss1), that functions in the translocation of newly synthesized secretory or membrane proteins into the ER for processing (Fig. 2a) (Itskanov and Park 2019). Additionally, Sss1 is a part of the Ssh1 complex comprised of Ssh1 (paralog of Sec61) and Sbh2 (paralog of Sbh1) that functions in protein translocation (Finke *et al.* 1996). Although not members of the Sec61 complex, Sec62, Sec63, Sec71, and Sec72 make up the Sec63 complex and similarly function in protein translocation. To assess if Ncr1 also interacts with the other members of the Sec61/Sbh1 and the Sec63 complex, prey protein plasmids were constructed and transformed into Ncr1-Cub-TF (Fig. 2b). Low stringency agar showed growth in all 3 cell dilutions for NubG-Sss1 (as expected), NubG-Sec61, NubG-Sbh1, NubG-Ssh1, and NubG-Sbh2, which together make up the Sec61/Ssh1 complex. No cell growth was seen in NubG-Sec62, NubG-Sec63, NubG-Sec71, and NubG-Sec72, which together make up the Sec63 complex. High stringency agar revealed blue colonies present in NubG-Sss1, NubG-Sec61, and NubG-Sbh1, while there was no cell growth in NubG-Ssh1 and NubG-Sbh2 suggesting a weaker interaction with these proteins. In a previous high-throughput yeast two-hybrid screen, Ncr1 was detected to interact with Sec63; this result however was not investigated further (Rizzolo *et al.* 2018). Therefore, Ncr1 interacts with the core Sec61/Sbh1 complex proteins (Sec61, Ssh1, Sbh1, and Sbh2) but not the Sec63 protein translocation complex (Sec62, Sec63, Sec71, and Sec72).

### Ncr1 interacts with Lip1 and Lag1 in the ceramide synthase complex

Lip1 functions as a member of the ceramide synthase complex (Lag1, Lac1, and Lip1) involved in the conversion of sphingoid bases into ceramide (Fig. 2c). To assess if Ncr1 also interacts with the other members of this complex (Lag1 and Lac1), prey plasmids for the expression of these proteins were constructed and transformed into Ncr1-Cub-TF (Fig. 2d). Low stringency agar showed cell growth in all 3 cell dilutions for NubG-Lip1 and NubG-Lag1, but not NubG-Lac1. High stringency agar resulted in no blue colony growth suggesting their interactions with Ncr1-Cub-TF are weak. Therefore, Ncr1 also interacts with Lag1, but not Lac1, which are members of the ceramide synthase complex.

### Oxygen-dependent sterol biosynthetic processes are required for the Ncr1-Cyb5, Ncr1-Lip1, Ncr1-Pga3, and Ncr1-Sss1 interactions

**Fig. 3.**
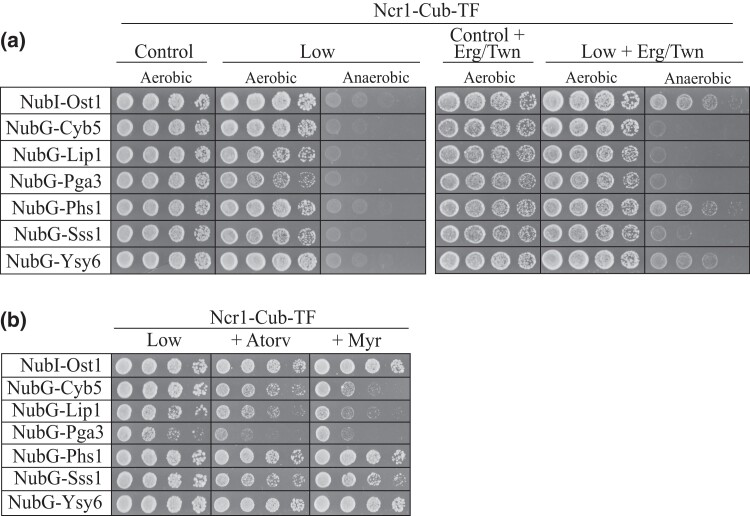
MYTH analysis in conditions of obligatory ergosterol uptake (anaerobiosis) and treatment with lipid biosynthesis inhibitors. a) Prey constructs [NubI-Ost1 (control), NubG-Cyb5, NubG-Lip1, NubG-Pga3, NubG-Phs1, NubG-Sss1, and NubG-Ysy6] were transformed into Ncr1-Cub-TF. Resulting transformants were grown overnight, serial diluted (1:5), and plated on selection stringency agar: control (SD-W), control + 20 µg/mL in ergosterol in 0.5% Tween 80 and 0.5% ethanol (Erg/Twn) (SD-W + Erg/Twn), low (SD-WH), and low + Erg/Twn (SD-WH + Erg/Twn) under aerobic (*de novo* ergosterol biosynthesis) and anaerobic (obligatory ergosterol uptake) conditions. Plates were incubated at 30°C for 5 days. Shown are cell dilutions 1:5, 1:25, 1:125, and 1:625. b) Resulting transformants were grown overnight, serial diluted (1:5), plated on selection agar: low (SD-WH) with vehicle control or supplemented with 7 µg/mL atorvastatin or 100 ng/mL myriocin. Plates were incubated at 30°C for 3 days. Shown are cell dilutions 1:5, 1:25, 1:125, and 1:625.

Yeast requires oxygen for *de novo* ergosterol biosynthesis, hence must utilize ergosterol uptake from the media for survival in anaerobic conditions (Andreasen and Stier 1953). Although NPC1 is known to function in uptake of exogenous cholesterol in mammalian cells (Hammond *et al.* 2019), Ncr1 in yeast has been shown to function in the uptake of sterols from lipid droplets in an anaerobic condition (Winkler *et al.* 2019). Deletion of either *NCR1* or *NPC2* results in numerous large lipid droplets deposited both around the vacuole and inside the vacuole, suggesting the cells internalize lipid droplets via Ncr1 at the vacuolar membrane but are unable to process/degrade the lipid droplets efficiently. Therefore, the MYTH interactions identified here were next tested under anaerobic conditions. As expected (Lorenz and Parks 1987), all yeast strains grown under anaerobic conditions without ergosterol supplementation showed no cell growth (Fig. 3a). Interestingly, when supplemented with ergosterol, cell growth of the MYTH constructs for NubG-Phs1 and NubG-Ysy6 were similar to the NubI-Ost1 control. In contrast, supplementation with ergosterol did not increase cell growth in MYTH constructs for NubG-Cyb5, NubG-Lip1, NubG-Pga3, and NubG-Sss1, suggesting that these interactions are perhaps required for ergosterol biosynthesis or another oxygen-dependent process.

### Lipid-modifying drugs reduce the strength of PPIs between Ncr1-Cyb5, Ncr1-Lip1, and Ncr1-Pga3

To further characterize the Ncr1 interactions in the context of lipid metabolism, MYTH analysis was conducted on media supplemented with lipid-regulating drugs that inhibit 2 different features of lipid metabolism, both of which are relevant to NP-C disease with mechanisms of action conserved from yeast to mammalian cells. Atorvastatin is an inhibitor of the first rate-limiting step of ergosterol biosynthesis (Chong and Seeger 1997) and myriocin is an inhibitor of the first and rate-limiting step of sphingolipid biosynthesis (Miyake *et al.* 1995). Relevance of the MYTH interactions to each of these aspects of lipid metabolism was measured via cell growth in the presence of these drugs compared to vehicle control. Interestingly, both atorvastatin and myriocin reduced cell growth of NubG-Cyb5, NubG-Lip1 and NubG-Pga3 (and marginally NubG-Sss1) relative to vehicle control on low stringency agar (Fig. 3b). Cell growth of NubG-Phs1 and NubG-Ysy6 appeared unaffected by treatment on low stringency agar. Therefore, physical interactions of Cyb5, Lip1, and Pga3 with Ncr1 were inhibited upon treatment with lipid-regulating drugs, atorvastatin, and myriocin.

### Deletion of *NCR1* and *CYB5* increases sensitivity to myriocin but not atorvastatin

**Fig. 4.**
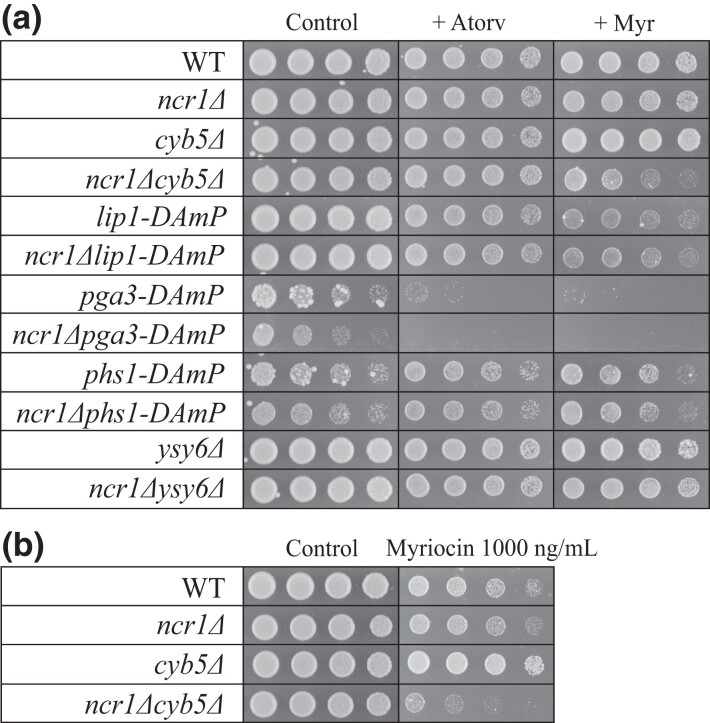
Cell growth response of single and double gene mutants to lipid biosynthesis inhibitors. Cells were grown overnight, serial diluted (1:5), and plated onto SC agar with or without atorvastatin (14 µg/mL) or myriocin (700 ng/mL) a) or myriocin (1000 ng/mL) b). Plates were incubated at 30°C for 2 days. Shown are cell dilutions 1:5, 1:25, 1:125, and 1:625.

Given PPIs with Ncr1 were lost under different lipid regulating conditions and treatments, the response (sensitivity or resistance) of single and double gene mutant strains to lipid regulating treatments was assessed (Fig. 4). Atorvastatin treatment in single and double deletion strains was used to determine strain sensitivity or resistance to ergosterol biosynthesis inhibition (Fig. 4a). Growth on control agar was reduced in both *pga3-DAmP* and *phs1-DAmP* strains relative to wild-type. Atorvastatin treatment (14 µg/mL) inhibited growth in the already weakened *pga3-DAmP* and *ncr1*Δ*pga3-DAmP* strains. No other mutant strain displayed reduced growth upon ergosterol biosynthesis inhibition through atorvastatin treatment. Myriocin treatment in single and double mutant strains was used to determine strain sensitivity or resistance to sphingolipid biosynthesis inhibition (Fig. 4a). Myriocin treatment (700 ng/mL) minimally reduced growth in both wild-type and *ncr1*Δ cells. Consistent with the literature (Fröhlich *et al.* 2015), growth of *cyb5*Δ cells appeared resistant to myriocin treatment even at 1000 ng/mL (Fig. 4b). In *ncr1*Δ*cyb5*Δ, growth was visibly reduced at 700 ng/mL suggesting deletion of *NCR1* in combination with the deletion of *CYB5* results in a myriocin sensitivity phenotype, not observed in the single deletion strains. Growth of *lip1-DAmP* and *ncr1*Δ*lip1-DAmP* was reduced relative to wild-type, while growth of *pga3-DAmP* was again completely inhibited. Interestingly, growth of treated *phs1-DAmP* appeared to again increase relative to untreated *phs1-DAmP* cells. Last, growth of *ysy6*Δ and *ncr1*Δ*ysy6*Δ was comparable to wild-type cells. Therefore, deletion of *NCR1* together with *CYB5* increased the double mutant sensitivity to myriocin but not atorvastatin suggesting a genetic interaction between these 2 genes is occurring to regulate sphingolipid perturbations.

### Deletion of *NCR1* and *CYB5* dysregulates sphingolipid biosynthesis

**Fig. 5.**
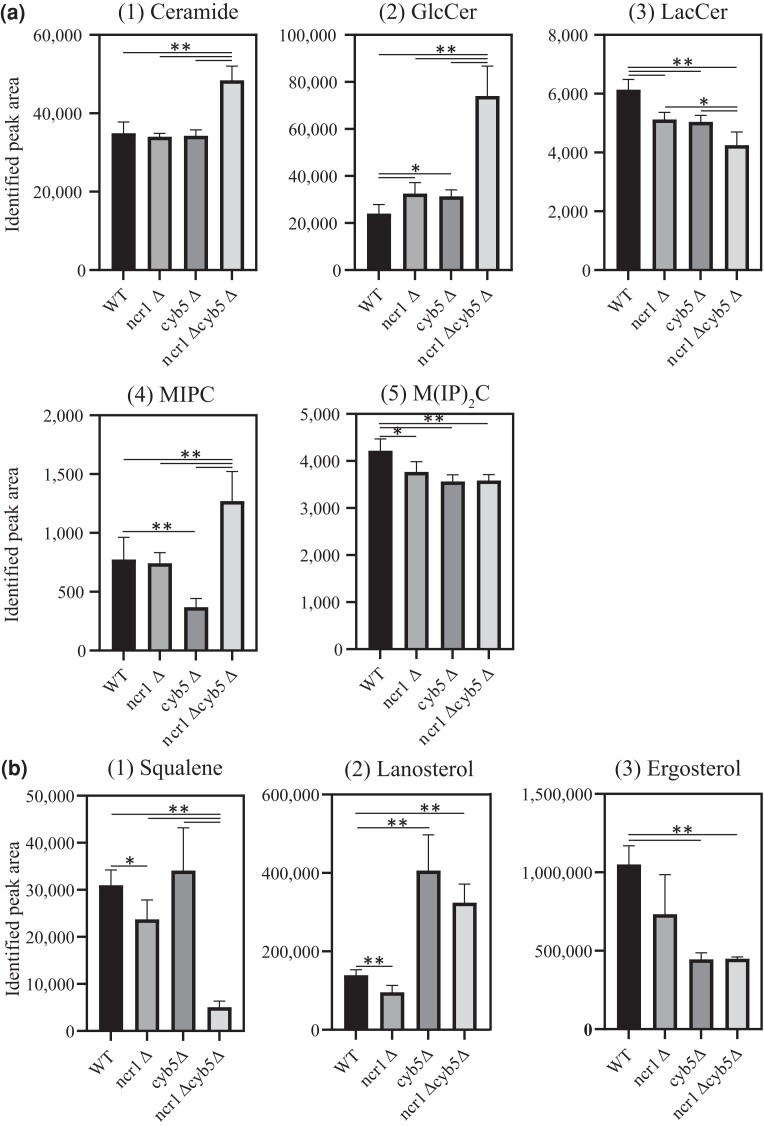
Mass spectrometry analysis of sphingolipids and sterols in *ncr1Δcyb5Δ* and single mutant strains. a) Polar lipids were extracted from 9.25 × 10^7^ cells (postdiauxic phase) using ethanol: ddH_2_0: diethylether: pyridine: ammonium hydroxide (15:15:5:1:0.018) and subjected to UHPLC-MS to quantify sphingolipid species: (1) ceramide, (2) glucosylceramide (GlcCer), (3) lactosylceramide (LacCer), (4) MIPC, and (5) M(IP)_2_C. b) Neutral lipids were extracted from 1.85 × 10^8^ cells (early-log phase) using MTBE:MeOH (10:3) and subjected to SFC-MS to quantify sterol lipid species: (1) squalene, (2) lanosterol, and (3) ergosterol. Lipids were normalized to total lipid. Mean ± SD from 6 biological replicates. *P < 0.05; **P < 0.01, Brown–Forsythe and Welch ANOVA test and corrected for using a Games–Howell's multiple comparisons test.

Deletion of *NCR1* and *NCR1* point mutants does not appear to dysregulate sterol synthesis and esterification (Malathi *et al.* 2004). These *NCR1* mutants do, however, show aberrations in sphingolipid biosynthesis (Malathi *et al.* 2004; Vilaça *et al.* 2014, 2018). Given *ncr1Δcyb5Δ* displayed sensitivity to myriocin (Fig. 4a), sphingolipids were measured using UHPLC-MS to see if the sphingolipid profile was different to wild-type (Fig. 5a.i–v). Interestingly, deletion of *NCR1* and *CYB5* resulted in a significant increase in ceramide levels relative to wild-type (*P = 0.0002*), *ncr1*Δ (*P = 0.0005*) and *cyb5*Δ (*P = 0.0002*). Glucosylceramide also significantly increased in *ncr1*Δ*cyb5*Δ relative to wild-type (*P = 0.0004)*, *ncr1*Δ (*P = 0.0009*) and *cyb5*Δ (*P = 0.0012*). A significant albeit less pronounced increase was also detected in *ncr1*Δ (*P = 0.029*) and *cyb5*Δ (*P = 0.018*) relative to wild-type. Lactosylceramide significantly decreased in *ncr1*Δ*cyb5*Δ relative to wild-type (*P < 0.0001*), *ncr1*Δ (*P = 0.0129*) and *cyb5*Δ (*P = 0.0217*). A significant reduction was also detected in *ncr1*Δ (*P = 0.0011*) and *cyb5*Δ (*P = 0.0006*) relative to wild-type. Consistent with the *NCR1Y_718_D* mutant (Malathi *et al.* 2004), mannose-inositolphosphoryl ceramide (MIPC) significantly increased in *ncr1*Δ*cyb5*Δ relative to wild-type (*P = 0.016*), *ncr1*Δ (*P = 0.0104*) and *cyb5*Δ (*P = 0.0007*). Deletion of *CYB5* alone resulted in a significant reduction in MIPC levels, relative to wild-type (*P = 0.009*) and *ncr1*Δ (*P < 0.0001*). Mannose-diinositolphosphoryl ceramide [M(IP)_2_C] significantly decreased in *ncr1*Δ*cyb5*Δ (*P = 0.0028*)*, ncr1*Δ (*P = 0.032*) and *cyb5*Δ (*P = 0.0022*) relative to wild-type. Assuming Ncr1 does not have another PPI to compensate for loss of Cyb5, and vice-versa, the deletion of *NCR1* and *CYB5* (the loss of the Ncr1-Cyb5 PPI) may lead to a block in the sphingolipid biosynthesis pathway—perhaps in the conversion of glycosylceramide to lactosylceramide and, as a result, ceramide levels are increased in the *ncr1*Δ*cyb5*Δ strain.

### Deletion of *CYB5* alone and deletion of *NCR1* and *CYB5* dysregulates ergosterol biosynthesis

The deletion of *CYB5* (in *Saccharomyces cerevisiae*) or *CybE* (in *Aspergillus fumigatus*) has previously been reported to result in decreased ergosterol and increased sterol intermediates (Rogers *et al.* 2004; Misslinger *et al.* 2017). Therefore, ergosterol and its precursors were measured to determine the effect of deleting both *NCR1* and *CYB5*, and if this would perhaps result in a NP-C-like phenotype. Supercritical fluid chromatography-mass spectrometry (SFC-MS) was performed on neutral lipid extracts of strains bearing *NCR1* and *CYB5* deletions (Fig. 5b.i–iii). Squalene levels were strikingly decreased in *ncr1Δcyb5Δ* relative to wild-type (*P < 0.0001*), *ncr1*Δ (*P = 0.0002)* and *cyb5*Δ (*P = 0.0019*). A significant reduction was also detected in *ncr1*Δ (*P = 0.0315*) relative to wild-type. Lanosterol levels significantly increased in *ncr1*Δ*cyb5*Δ relative to wild-type (*P = 0.0004*). Consistent with findings by Rogers *et al.* (2004), deletion of *CYB5* alone resulted in a significant increase in lanosterol levels, relative to wild-type (*P = 0.0028)*, while deletion of *NCR1* resulted in decreased levels of lanosterol relative to wild-type (*P = 0.004*). Last, ergosterol levels were significantly decreased in *ncr1*Δ*cyb5*Δ relative to wild-type (*P = 0.0002*). A significant reduction was also detected in *cyb5*Δ relative to wild-type (*P < 0.0001*). Therefore, although deletion of *NCR1* and *CYB5* does not appear to give the typical NP-C hallmark phenotype of ergosterol accumulation, the loss of these genes contributes to a block in squalene synthesis, upstream of ergosterol, perhaps via redundant functions of Ncr1 and Cyb5 in squalene synthesis.

### Deletion of *NCR1* and *CYB5* affects cell viability

**Fig. 6.**
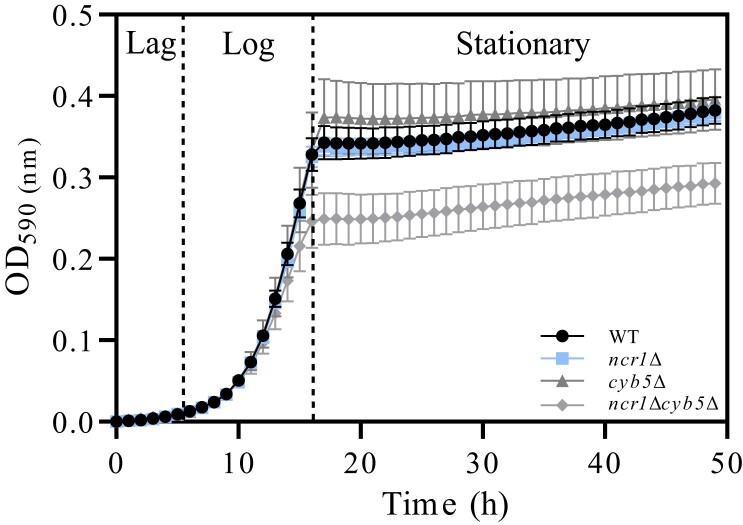
Cell growth defect of *ncr1Δcyb5Δ* over a 48-h period. Strains were grown in selection media (SC, SC + clonNAT, SC + G418, or SC + clonNAT + G418) overnight and diluted to 0.128 × 10^7^ cells/mL before hourly reads for 48 h using the Envision 2102 Multilabel plate reader (Perkin Elmer) at 590 nm. Data shown are mean ± SEM.

To determine if single and double mutants affected cell viability, cell density was measured hourly over a 48-h period (Fig. 6). Because double mutant strains were successfully constructed, this suggests that *NCR1* and *CYB5* are not synthetic lethal. This leaves open the possibility for a synthetic sick interaction, where 2 separately nonlethal mutations result in reduced growth. Typically, yeast cell growth goes through 3 phases: first, a lag phase where cells become acclimated to the new environment; log-phase follows where cells continually proliferate and increase exponentially with time; once glucose is consumed, cells undergo a diauxic shift and cell proliferation slows until cells enter a stationary phase and cease proliferation (Gray *et al.* 2004). From 18 to 48 h, cell growth is significantly reduced in *ncr1*Δ*cyb5*Δ relative to wild-type and *ncr1*Δ (*P < 0.05*) and close to significance (*P = 0.06*) relative to *cyb5*Δ. Therefore, growth of the *ncr1*Δ*cyb5*Δ strain appears stunted, with the culture plateauing at a similar time-point to wild-type and single deletions, but at a lower cell density, therefore reflecting a negative genetic interaction between *NCR1* and *CYB5*.

### Ncr1 and Cyb5 colocalize at the ER-vacuole membrane interface

**Fig. 7.**
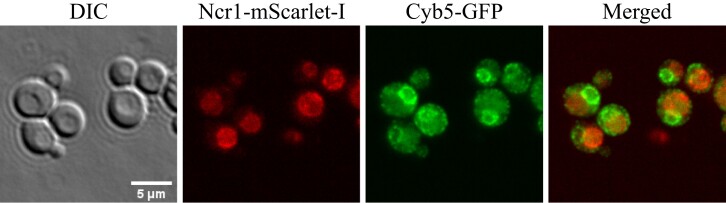
Colocalization of Ncr1 and Cyb5 at the ER-vacuole membrane interface. Cells expressing Ncr1-mScarlet-I and Cyb5-GFP were grown in SC from a concentration of 0.255 × 10^7^ cells/mL to 1.26 × 10^7^ cells/mL and imaged using an IN Cell Analyzer 6500 with a 60 × objective lens.

To further investigate the Ncr1-Cyb5 PPI identified above using MYTH, we next sought to gain subcellular context of this PPI. Using a strain expressing Ncr1-mScarlet and Cyb5-GFP, localization of each protein was monitored using confocal microscopy. As expected (Malathi *et al.* 2004; Cherry *et al.* 2012), Ncr1-mScarlet was localized to the vacuolar membrane and Cyb5-GFP was localized to the ER membrane, while the merged image revealed colocalization of the 2 proteins at the interface between the ER and vacuolar membranes (Fig. 7). This result supports our discovery of the Ncr1-Cyb5 PPI and suggests this PPI may be active at the ER-vacuole membrane interface.

### Alanine mutagenesis of *CYB5* reveals possible sites of interaction between Ncr1-Cyb5

**Fig. 8.**
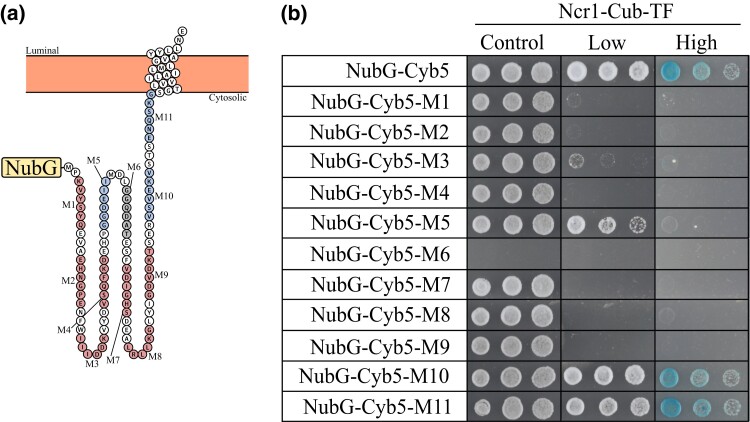
Several Cyb5 mutants do not interact with Ncr1 via MYTH analysis, revealing possible sites of interaction. a) Predicted topology of Cyb5 with 11 mutants highlighted [mutant 1 (M1)–mutant 11 (M11)] (Omasits *et al*. 2014). Red circles, no MYTH interaction; blue circles, MYTH interaction; gray circles, no result. b) All Cyb5 constructs (NubG-Cyb5, NubG-Cyb5 M1, NubG-Cyb5 M2, NubG-Cyb5 M3, NubG-Cyb5 M4, NubG-Cyb5 M5, NubG-Cyb5 M6, NubG-Cyb5 M7, NubG-Cyb5 M8, NubG-Cyb5 M9, NubG-Cyb5 M10, and NubG-Cyb5 M11) were transformed into Ncr1-Cub-TF. Resulting transformants were grown overnight, serial diluted (1:5), and plated on selection stringency agar [control (SD-W), low (SD-WH), high (SD-WH + X-Gal)] and incubated at 30°C for 3 days. Shown are cell dilutions 1:5, 1:25, and 1:125.

To characterize possible sites of interaction, we performed alanine mutagenesis of the prey proteins; this approach has been effectively used to identify residues required for PPIs (Cunningham and Wells 1989; Lefèvre *et al.* 1997). Mutants were designed using the predicted topology of each protein as a guide (Omasits *et al.* 2014). Cytosolic protruding segments of amino acid stretches (5–6 aa) were changed to alanine and cloned into the pPR3N plasmid for N-terminal tagging. Eleven Cyb5 mutants were designed, constructed, and named M1-M11 (Fig. 8a). Wild-type and mutagenized Cyb5 in the pPR3N vector were transformed into Ncr1-Cub-TF and MYTH analysis conducted. Figure 8b shows equivalent cell growth on control agar, excluding mutant 6 (M6), indicating all transformations with Ncr1-Cub-TF but M6 were successful and cell density across samples was comparable. The inability of M6 to survive upon transformation into Ncr1-Cub-TF suggests a possible toxicity—this however was not further investigated. In contrast, cell growth on low stringency agar appeared only in wild-type Cyb5, M5, M10, and M11. High stringency agar contained cell growth in wild-type Cyb5, M10, and M11. These results indicate that M1, M2, M3, M4, M6, M7, M8, and M9 regions in Cyb5 are required for the Ncr1-Cyb5 interaction. Alternatively, these regions may be important for protein expression and/or ER membrane localization of Cyb5, whereby mutations in these regions would prevent its interaction with Ncr1.

## Discussion

The yeast model of NP-C disease has been useful for identifying candidate therapeutic drugs that have been translated to animal models and human clinical trials as well as better understanding the basic science of lipid transport in eukaryotes (Munkacsi *et al.* 2011). Previously reported phenotypes of the yeast NPC model include defects in sphingolipid metabolism (Malathi *et al.* 2004; Vilaça *et al.* 2014, 2018), lipid droplet uptake and processing (Tsuji *et al.* 2017; Winkler *et al.* 2019), and lowered vacuolar pH (Brett *et al.* 2011). No study yet has associated a PPI with any of these phenotypes in yeast. We hypothesized proteins interacting with Ncr1 may help to identify orthologous proteins that function alongside mammalian NPC1 and NPC2 in cholesterol homeostasis at the LE/Ly or alternatively identify new functions of the Ncr1/NPC1 protein. Here, we used a proteome-wide MYTH analysis to identify 6 full-length proteins physically interacting with Ncr1, of which 3 were confirmed by co-IP. We further characterized the Ncr1-Sss1 and Ncr1-Lip1 via MYTH analyses of other proteins in the Sss1 (Sec61 complex) and Lip1 (ceramide synthase) complexes. Notably, we report for the first time an NP-C disease phenotype (glucosylceramide accumulation) that is consequence of the loss of a PPI (via gene deletions of *NCR1* and *CYB5*).

Prior to this study, a physical or genetic interaction between Ncr1/*NCR1* and Cyb5/*CYB5* has not been documented. The deletion of *CYB5* (*cyb5*Δ) (in *S. cerevisiae*) or *CybE* (in *A. fumigatus*) has previously been reported to result in decreased ergosterol and increased sterol intermediates (Rogers *et al.* 2004; Misslinger *et al.* 2017). Similar to previous findings (Malathi *et al.* 2004), deletion of *NCR1* (*ncr1*Δ) does not result in an accumulation of ergosterol (cholesterol in mammals), which is the hallmark phenotype in NP-C diseased patients (Vanier 2010; Hammond *et al.* 2019). Yeast, however, lacks LDL receptors required for sterol uptake, instead transfer of exogenous ergosterol occurs through Aus1 and Pdr11 (Wilcox et al. 2002); therefore, Ncr1 may undertake a different primary function in the yeast model. In mammals, cytochrome b5 is thought to function as an electron donor in the conversion of dimethylzymosterol to zymosterol (by sterol 4α-methyl oxidase) and in the conversion of 7-dehydocholesterol to cholesterol (by sterol C5-desaturase) (Porter *et al.* 2010; Giese *et al.* 2015). Interestingly, in NP-C disease, 7-ketocholesterol, a by-product of 7-dehydrocholesterol, is elevated in NP-C diseased patients and has been used as a biomarker for NP-C disease (Porter *et al.* 2010).

Several steps in ergosterol biosynthesis require oxygen and heme/iron, which directly associate with Erg11/Erg5, and Erg25/Erg3, as a cytochrome b5 cofactor (Jordá and Puig 2020). Iron deficiency reduces metabolic flux in sterol biosynthesis, leading to a decrease in ergosterol and zymosterol, and an accumulation of Erg1 and Erg11 substrates, squalene and lanosterol, respectively (Shakoury-Elizeh *et al.* 2010). In relation to NP-C disease, iron levels are dysregulated in NP-C disease mice, showing an increase in the brain and a decrease in the liver relative to wild-type (Hung *et al.* 2020). In yeast, when heme/iron levels are low, the function of enzymes like Erg11 are likely reduced leading to an accumulation of lanosterol; this accumulation then feeds back and inhibits Erg1, leading to an increase in squalene levels. It is proposed that we see a similar result with deletion of *CYB5* whereby deletion of this heme-binding electron transfer system leads to inefficient Erg11 function and subsequent accumulation of lanosterol and reduction of ergosterol. Upon double deletion of *NCR1* and *CYB5* (*ncr1*Δ*cyb5*Δ), squalene is significantly reduced relative to wild-type and single deletion strains (Fig. 5). This striking reduction in squalene suggests a block in the conversion of Farnesyl-PP to squalene in the ergosterol biosynthesis pathway. Farnesyl-PP has several branch points, where 2 molecules are required for conversion into squalene, or alternatively, branched into other biosynthetic pathways for the production of ubiquinone, dolichol, prenylated proteins and heme (Jordá and Puig 2020). Although not well documented, the formation of Farnesyl-PP has been suggested to occur in the vacuole (Jordá and Puig 2020). Perhaps, *NCR1* is involved in the transfer of sterol intermediates, and in the case where *NCR1* is deleted, it cannot be taken up by the vacuole and further processed into Farnesyl-PP for conversion into squalene.

Rather than sterol accumulation, it was previously hypothesized that the primordial toxic metabolite was sphingolipids; this was based on MIPC accumulation and sensitivity to 2 sphingolipid biosynthesis inhibitors (Aureobasidin A and Australifungin) in the *NCR1Y_718_D* mutant (Malathi *et al.* 2004). Our results for *ncr1*Δ*cyb5*Δ recapitulate this MIPC accumulation and in addition we show glucosylceramide accumulation, which is typical in NP-C patients (Vanier 2010). These results suggest that perhaps the *NCR1* 718 aa is critical to the Ncr1-Cyb5 PPI. Through alanine mutagenesis, 11 Cyb5 mutants were designed and constructed. Interestingly, interactions with Ncr1 were inhibited in M1-M4 and M7-M9. Interactions remained in full-length, M5, M10, and M11, suggesting that the cytoplasmic protruding region of Cyb5 is important for a physical interaction with Ncr1. Cyb5 has one functional domain—the cytochrome b5 heme-binding domain (2–78 aa). Nearly all strains constructed with mutations within the cytochrome b5 heme-binding domain, resulted in the interaction being inhibited. The cytochrome b5 heme-binding domain is a region seen in several other proteins including Cyb2, Irc21, Scs7, Ole1, and Dap1 (Xia and Mathews 1990; Mitchell and Martin 1995, 1997; Mallory *et al.* 2005), many of which function in lipid metabolism. The Ncr1-Cyb5 PPI appears to be fundamental to the yeast model of NP-C disease. Without the PPI (via gene deletions), glucosylceramide accumulates, suggesting the PPI is critical to sphingolipid metabolism in NP-C pathology and healthy cells overall.

Liquid growth curve analysis of single and double mutant strains revealed a stunted and statistically reduced growth (optical density) in *ncr1*Δ*cyb5*Δ cells. This stunted growth phenotype in *ncr1*Δ*cyb5*Δ is present at log-phase whereby exponential growth is slowed, diauxic shift from fermentation to respiration occurs earlier than control, and the culture arrests in growth entering the G0 state at a significantly lower cell density than control. This growth pattern is typical in cells missing essential nutrients such as nitrogen, phosphate, or glucose (Gray *et al.* 2004; Smets *et al.* 2010; Ramos-Gomez *et al.* 2017). Single mutant strains *ncr1*Δ and *cyb5*Δ each displayed a growth curve phenotype similar to that of wild type, which was consistent with previously reported results (Truan *et al.* 1994; Malathi *et al.* 2004). Conversely, deletion of *CybE* in *A. fumigatus* displays a severe growth phenotype with a small, compacted colony with poor conidiation on agar (Misslinger *et al.* 2017; Zhang *et al.* 2021). Although cell growth of *ncr1*Δ*cyb5*Δ on agar was not visually impaired, a clear disruption in growth upon deletion of these 2 genes is intriguing, and questions what essential nutrients these cells are lacking, or conversely, what metabolic intermediates are perhaps accumulating, causing this irregularity in growth relative to control.

Lip1 is an essential protein in the ceramide synthase complex with the nonessential proteins Lag1 and Lac1 (Guillas *et al.* 2001; Schorling *et al.* 2001; Vallée and Riezman 2005). Lip1 has no mammalian ortholog; however, Lag1, Lac1, and ceramide synthase are conserved across eukaryotes. Ceramide synthase functions in the conversion of sphingoid bases into ceramide, DHS to dihydroceramide, or PHS to phytoceramide in sphingolipid biosynthesis (Megyeri *et al.* 2019). Up until recently, Lag1 and Lac1 were thought to be homologs with identical functions as ceramide synthases; however, Lag1 and Lac1 appear to have distinct substrate specificity. Lag1 preferentially synthesizes phytosphingolipids, while Lac1 preferentially synthesizes dihydrosphingolipids. Interestingly, *LAG1* expression is upregulated and phytoceramides are increased in cells where *NCR1* is deleted (Vilaça *et al.* 2018).This observation is fitting, given Lag1 preferentially synthesizes phytosphingolipids (Megyeri *et al.* 2019). Given the genetic interaction result by Vilaça *et al.* (2018) and the physical interactions of Lip1 and Lag1 with Ncr1 identified in this study, a connection between Ncr1 and ceramide synthase is likely critical to better understand the yeast model of NP-C disease.

Protein translocation is highly conserved in eukaryotic cells and is essential for the biogenesis of transmembrane and secretory proteins (Linxweiler *et al.* 2017). The exact process of how Ncr1 is processed before localization to the vacuolar membrane (or NPC1 at the lysosome in mammals) is not fully understood. It is feasible that Ncr1 passes through the Sec61 core complex after translation and prior to protein folding and translocation to the vacuolar membrane given that both NPC1 and Ncr1 contain a Sec-specific signal peptide at their N-terminus responsible for targeting the newly synthesized protein to the ER for posttranslation processing (MNVLWIIALVGQLMRLVQG). The Sec61 complex has also been observed to function as a channel for the passive efflux of Ca^2+^ ions from inside the ER to the cytosol (Wirth *et al.* 2003; Van Coppenolle *et al.* 2004; Lang *et al.* 2011). Interestingly, increased calcium levels in the ER correct the folding of NPC1 protein and rescues the defective trafficking of cholesterol and sphingolipids. Independently, treatment of NPC-mutant mouse cells and diseased mice with curcumin, a sarcoplasmic/ER Ca^2+^ ATPase antagonist that functions in the active import of Ca^2+^ into the ER lumen, was also therapeutic via normalization of sphingolipid levels in these models (Lloyd-Evans *et al.* 2008). The role of Sec61 in these therapeutic rescues has not been investigated. It is plausible that the NPC1-SEC61 PPI is upregulated and a contributor to this therapeutic rescue, albeit it remains to be determined whether this occurs via improved folding, increased ER calcium, or both.

Although not as well studied, Ysy6, one of the strongest PPIs identified in this study, has previously been found to be associated with Sec61. Ribosome-associated membrane protein 4 (RAMP4), also known as stress associated endoplasmic reticulum protein 1 (SERP1), is the mammalian ortholog of Ysy6, that when under ER stress, RAMP4 stabilizes newly synthesized membrane proteins associating with the Sec61 complex (Sakaguchi *et al.* 1991; Yamaguchi *et al.* 1999). Given that bacterial and mammalian orthologs of Ysy6 (i.e. RAMP4) stabilize membrane proteins, such as the Sec proteins, during stress (Sakaguchi *et al.* 1991; Yamaguchi *et al.* 1999) and that ER stress is prominent in NP-C disease (Vázquez *et al.* 2012), Ysy6 may have a similar function and therefore, under this function, it may interact with NPC1/Ncr1. Loss of this interaction may occur during NP-C disease and more specifically, NPC1 mutations in sites that govern this PPI.

In summary, our results gained using MYTH to identify PPIs for Ncr1 reiterate the importance of using a different methodology to identify PPIs. Our results identify PPIs that may have been suspected (e.g. Ncr1 interacting with the Sec61 translocation complex), results that are consistent with previously identified genetic interactions (e.g. Ncr1 interacting with Lag1/Lip1 in the ceramide synthase complex), and unsuspected interactions (e.g. Ncr1 with cytochrome b5). Ncr1/NPC1 may function with cytochrome b5, an electron donor, at different stages of lipid metabolism—this could help explain why NP-C disease results in the accumulation of several lipid species. Although pathways/complexes highlighted in this work are conserved in mammals, it is necessary to test whether the same physical interactions are observed between the ceramide synthase complex (LAG1 and LAC1 in mammals), the Sec61 protein translocation complex (SEC61α, SEC61β, and SEC61γ), and CYB5B. This work has utilized the simple and informative model organism yeast to highlight pathways to better understand NP-C disease and lipid transport.

## Supplementary Material

iyad129_Supplementary_Data

## Data Availability

All data are contained in the manuscript herein. Supplemental material available at GENETICS online.
