# Supplementary material for: Identification and characterization of protein interactions with the major Niemann–Pick type C disease protein in yeast reveals pathways of therapeutic potential
Source: Genetics. 2023 Jul 13;225(1):iyad129. doi: 10.1093/genetics/iyad129 (PMC10471228; doi:10.1093/genetics/iyad129)
Supplement: iyad129_Supplementary_Data [file iyad129_supplementary_data.zip › Figure_S1_GENETICS-2023-306236.pdf]

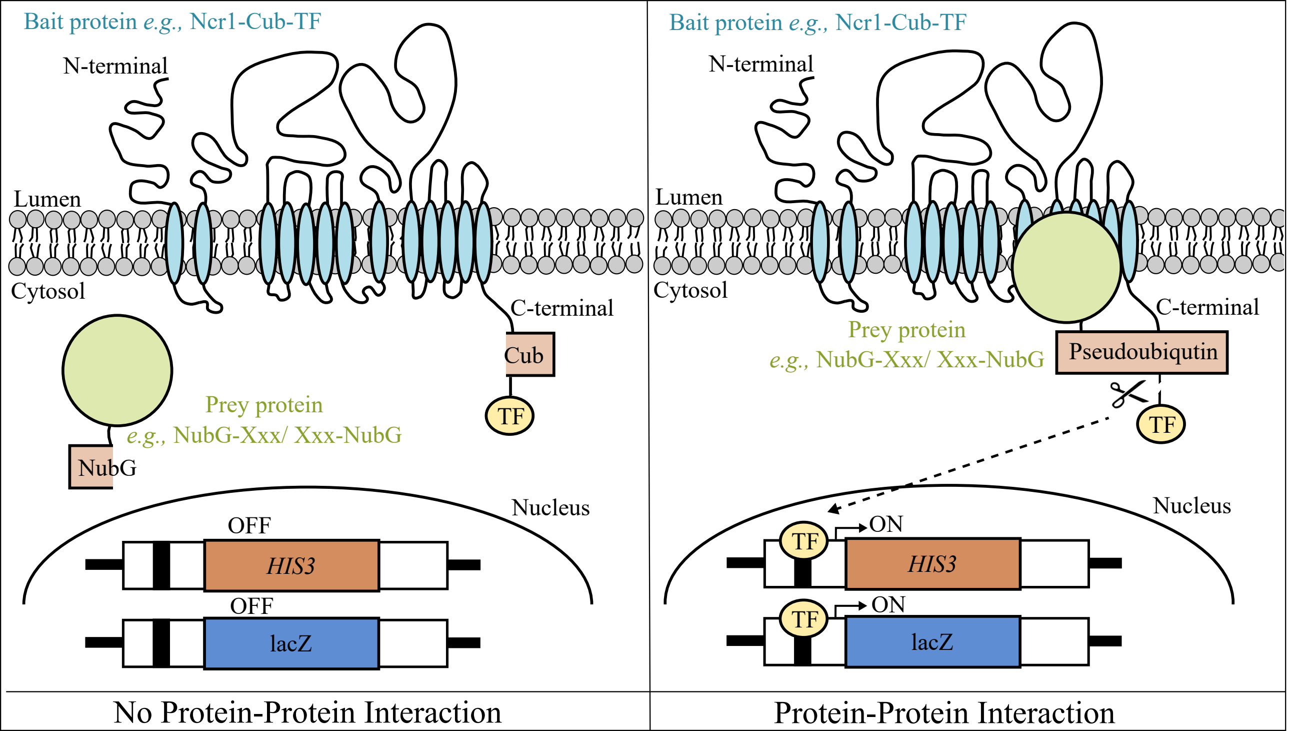
Bait protein e.g., Ncr1-Cub-TF
N-terminal
Lumen
Cytosol
C-terminal
Cub
TF
NubG
Prey protein
e.g., NubG-Xxx/ Xxx-NubG
Nucleus
OFF
HIS3
OFF
lacZ
No Protein-Protein Interaction
Bait protein e.g., Ncr1-Cub-TF
N-terminal
Lumen
Cytosol
C-terminal
Prey protein
e.g., NubG-Xxx/ Xxx-NubG
Pseudoubiqutin
TF
Nucleus
TF
ON
HIS3
TF
ON
lacZ
Protein-Protein Interaction
