# Supplementary material for: Identification and characterization of protein interactions with the major Niemann–Pick type C disease protein in yeast reveals pathways of therapeutic potential
Source: Genetics. 2023 Jul 13;225(1):iyad129. doi: 10.1093/genetics/iyad129 (PMC10471228; doi:10.1093/genetics/iyad129)
Supplement: iyad129_Supplementary_Data [file iyad129_supplementary_data.zip › Supplemental_Figure_Legends_GENETICS-2023-306236.docx]

Figure S1: **MYTH overview.** MYTH is a variant of the Y2H assay developed specifically for detecting PPI occurring with membrane proteins (Snider et al., 2010). MYTH is based around split ubiquitin, where ubiquitin is split into two portions, N-terminal (NubG) and C-terminal (Cub) (light orange). Proteins of interest, bait (light blue) and prey (light green), are fused to Cub and NubG respectively. Additionally, a transcription factor (TF, yellow) is fused to Cub. If bait and prey interact (right column), this brings Cub and NubG in close proximity forming a pseudoubiquitin molecule leading to activation of ubiquitin-specific proteases (scissors symbol) and subsequent cleavage of the TF that once inside the nucleus activates specific reporter gene/s, *HIS3* (dark orange) and *LacZ* (blue). If bait and prey do not interact (left column), the pseudoubiquitin molecule does not form, the TF is not cleaved, and thus the reporter gene(s) are not activated.

Figure S2. **Ncr1-Cub-TF bait verification.** A. To determine if Ncr1-Cub-TF self-activates, NubI-Ost1 (positive control) and NubG-Ost1 (negative control) were transformed into the Ncr1-Cub-TF strain. Serial dilutions (1:5) of saturated cultures were plated on selection stringency agar: control (SD-W), low (SD-WH), high (SD-WH + X-Gal) and incubated at 30°C for 3 days. Shown are three cell dilutions 1:5, 1:25 and 1:125. B. To determine correct localisation at the vacuolar membrane, *NCR1* was tagged with Cub-TF-YFP using the L3 plasmid. Resulting transformants were grown overnight in SC + G418 and subcultured for 5 hours in YPD. Cells were imaged on an IN Cell Analyzer 6500 (60X objective lens, DIC (100 ms exposure), green filter (1 s exposure)).

Figure S3: **Full-length prey protein plasmid construction by homologous recombination.** Full-length, N-terminally tagged prey constructs were generated using *in vivo* homologous recombination as previously described (Ma *et al.* 1987). A. Gene-specific primers included the start codon and 20-25 bp downstream as well as the stop codon and 20-25 bp upstream, each paired with a 40 bp sequence homologous to the multiple cloning site (MCS) and terminator of the prey plasmid. B. Prey plasmid was linearized at the MCS using SfiI. C. Co-transformation of the PCR product and linearized plasmid into MYTH reporter strain via homologous recombination. D. Plasmid extracted from yeast, transformed into bacteria, and propagated before extraction and subsequent MYTH analysis. *XXX* ORF of gene of interest (green); CYC1 (grey) promoter; NubG (orange) MYTH prey tag; MCS (pink); CYC1 (grey) terminator.
