# Supplementary material for: Identification and characterization of protein interactions with the major Niemann–Pick type C disease protein in yeast reveals pathways of therapeutic potential
Source: Genetics. 2023 Jul 13;225(1):iyad129. doi: 10.1093/genetics/iyad129 (PMC10471228; doi:10.1093/genetics/iyad129)
Supplement: iyad129_Supplementary_Data [file iyad129_supplementary_data.zip › Tables_S1-S2_GENETICS-2023-306236.pdf]

SUPPLEMENTARY

Table S1: **Primers used to construct MYTH strains.** Non-italicized letters are gene-specific and italicized letters are plasmid-specific.

Ncr1-Cub construct-F:
TTTGGTGGTGAAAGCTATAGGGACGATTCCATCGAAGCAGAAGAT*ATGTCGGGGGGGATCCCTCC*

Ncr1-Cub construct-R:
TTACCTATTTTTTCACTACGTAAAATATAGTATAATCTGCTATGG*ACTATAGGGAGACCGGCAGA*

Ncr1-Cub verify-F:
TGTGTACTTGCATTCGCCCAATCGA

kanMX L2/L3 verify-R:
GAGCGTTTCCCTGCTCGCAG

L2 verify sequencing-R:
GCCGTTAACGCTTTCATGC

L3 verify sequencing-R:
TTGTGCCCATTAACATCACC

NubG-Cyb5 construct-F:
*ATCCAAGCAGTGGTATCAACGCAGAGTGGCCATTACGGCC*ATGCCTAAAGTTTACAGTTACCA

NubG-Cyb5 construct-R:
*TACATGACTCGAGGTCGACGGTATCGATAAGCTTGATATC*TTATTCGTTCAACAAATAATAAG

NubG-Lip1 construct-F:
*ATCCAAGCAGTGGTATCAACGCAGAGTGGCCATTACGGCC*ATGTCTCAACCCACTCCCATCAT

NubG-Lip1 construct-R:
*TACATGACTCGAGGTCGACGGTATCGATAAGCTTGATATC*TCACATGTGATAAATTGTGGAAT

NubG-Mhf1 construct-F:
*ATCCAAGCAGTGGTATCAACGCAGAGTGGCCATTACGGCC*ATGAATGACGATGAAGATAGAGC

NubG-Mhf1 construct-R:
*TACATGACTCGAGGTCGACGGTATCGATAAGCTTGATATC*TTATTCTTGAGTAACTCTTTCCT

NubG-Pga3 construct-F:
*ATCCAAGCAGTGGTATCAACGCAGAGTGGCCATTACGGCC*ATGTCAAAAGAAGACATAGAAGG

NubG-Pga3 construct-R:
*TACATGACTCGAGGTCGACGGTATCGATAAGCTTGATATC*TTAAAAGACGAAGACTTGATCGT

NubG-Phs1 construct-F:
*ATCCAAGCAGTGGTATCAACGCAGAGTGGCCATTACGGCC*ATGTCAAAAAAACTTGCGTCACC

NubG-Phs1 construct-R:
*TACATGACTCGAGGTCGACGGTATCGATAAGCTTGATATC*TCAAATTAGTTTCTTCCCGAAAG

NubG-Snf1 construct-F:
*ATCCAAGCAGTGGTATCAACGCAGAGTGGCCATTACGGCC*ATGAGCAGTAACAACAACACAAA

NubG-Snf1 construct-R:
*TACATGACTCGAGGTCGACGGTATCGATAAGCTTGATATC*TCAATTGCTTTGACTGTTAACGG

NubG-Sss1 construct-F:

*ATCCAAGCAGTGGTATCAACGCAGAGTGGCCATTACGGCC*ATGGCTAGAGCTAGTGAAAAAGG

NubG-Sss1 construct-R:
*TACATGACTCGAGGTCGACGGTATCGATAAGCTTGATATC*TTAAACAATAACGTATCTGATTG

NubG-Vba1 construct-F:
*ATCCAAGCAGTGGTATCAACGCAGAGTGGCCATTACGGCC*ATGCAAACACTAGACGAGACTTC

NubG-Vba1 construct-R:
*TACATGACTCGAGGTCGACGGTATCGATAAGCTTGATATC*TCAAGAACTTGAACTACGTTTGT

NubG-Vma3 construct-F:
*ATCCAAGCAGTGGTATCAACGCAGAGTGGCCATTACGGCC*ATGACTGAATTGTGTCCTGTCTA

NubG-Vma3 construct-R:
*TACATGACTCGAGGTCGACGGTATCGATAAGCTTGATATC*TTAACAGACAACATCTTGAGTAG

NubG-Vma9 construct-F:
*ATCCAAGCAGTGGTATCAACGCAGAGTGGCCATTACGGCC*ATGAGTAGTTTGTATGTGTCATA

NubG-Vma9 construct-R:
*TACATGACTCGAGGTCGACGGTATCGATAAGCTTGATATC*TTATTCTGCAAATTCAGGTCTCA

NubG-Vma11 construct-F:
*ATCCAAGCAGTGGTATCAACGCAGAGTGGCCATTACGGCC*ATGTCAACGCAACTCGCAAGTAA

NubG-Vma11 construct-R:
*TACATGACTCGAGGTCGACGGTATCGATAAGCTTGATATC*TCATTCAGAGCCTCTAGTGTTCA

NubG-Voa1 construct-F:
*ATCCAAGCAGTGGTATCAACGCAGAGTGGCCATTACGGCC*ATGGTGTTCGGTCAGCTGTATGC

NubG-Voa1 construct-R:
*TACATGACTCGAGGTCGACGGTATCGATAAGCTTGATATC*TTAATTGTTTTTTTTTATTGGGT

NubG-Ysy6 construct-F:
*ATCCAAGCAGTGGTATCAACGCAGAGTGGCCATTACGGCC*ATGGCCGTACAGACACCAAGACA

NubG-Ysy6 construct-R:
*TACATGACTCGAGGTCGACGGTATCGATAAGCTTGATATC*TCATAGGATATAGCTGATTAGTT

NubG-Sbh1 construct-F:
*ATCCAAGCAGTGGTATCAACGCAGAGTGGCCATTACGGCC*ATGTCAAGCCCAACTCCTCCAGG

NubG-Sbh1 construct-R:
*TACATGACTCGAGGTCGACGGTATCGATAAGCTTGATATC*TTAAAATAACTTACCGGCAACTT

NubG-Sec61 construct-F:
*ATCCAAGCAGTGGTATCAACGCAGAGTGGCCATTACGGCC*ATGTCCTCCAACCGTGTTCTAGA

NubG-Sec61 construct-R:
*TACATGACTCGAGGTCGACGGTATCGATAAGCTTGATATC*TCACATCAAATCAGAAAATCCTG

NubG-Sec62 construct-F:
*ATCCAAGCAGTGGTATCAACGCAGAGTGGCCATTACGGCC*ATGTCAGCCGTAGGTCCAGGTAG

NubG-Sec62 construct-R:
*TACATGACTCGAGGTCGACGGTATCGATAAGCTTGATATC*TCAGTTTTGTTCGGCTTTTTCAT

NubG-Sec63 construct-F:

*ATCCAAGCAGTGGTATCAACGCAGAGTGGCCATTACGGCC*ATGCCTACAAATTACGAGTATGA

NubG-Sec63 construct-R:
*TACATGACTCGAGGTCGACGGTATCGATAAGCTTGATATCC*TATTCTGGTGATTCATCATCTT

NubG-Sec71 construct-F:
*ATCCAAGCAGTGGTATCAACGCAGAGTGGCCATTACGGCC*ATGTCCGAATTTAATGAAACAAA

NubG-Sec71 construct-R:
*TACATGACTCGAGGTCGACGGTATCGATAAGCTTGATATC*CTAATTGACTAATCTTCCATCAT

NubG-Sec72 construct-F:
*ATCCAAGCAGTGGTATCAACGCAGAGTGGCCATTACGGCC*ATGGTTACCCTTGAATACAATGC

NubG-Sec72 construct-R:
*TACATGACTCGAGGTCGACGGTATCGATAAGCTTGATATC*TTATTCACCGTTATATTCGGCCA

NubG-Ssh1 construct-F:
*ATCCAAGCAGTGGTATCAACGCAGAGTGGCCATTACGGCC*ATGTCTGGTTTTCGTCTAATTGA

NubG-Ssh1 construct-R:
*TACATGACTCGAGGTCGACGGTATCGATAAGCTTGATATC*TTACATAGCACCTGGAACACCCA

NubG-Sbh2 construct-F:
*ATCCAAGCAGTGGTATCAACGCAGAGTGGCCATTACGGCC*ATGGCAGCTTCAGTTCCACCAGG

NubG-Sbh2 construct-R:
*TACATGACTCGAGGTCGACGGTATCGATAAGCTTGATATC*TTATATAATGTGTGTAAATTTCG

NubG-Lag1 construct-F:
*ATCCAAGCAGTGGTATCAACGCAGAGTGGCCATTACGGCC*ATGACATCAGCTACGGACAAATC

NubG-Lag1 construct-R:
*TACATGACTCGAGGTCGACGGTATCGATAAGCTTGATATC*TTATTCACACTTTTCCTTAGATT

NubG-Lac1 construct-F:
*ATCCAAGCAGTGGTATCAACGCAGAGTGGCCATTACGGCC*ATGTCGACAATAAAGCCAAGCCC

NubG-Lac1 construct-R:
*TACATGACTCGAGGTCGACGGTATCGATAAGCTTGATATC*TCAAATATCCTTTTTCGTTGGAG

NubG-verify-seq-F:
GTCAAGACTTTGACCGG

Table S2: **Sequences used in alanine mutagenesis analysis.** Lowercase sequence represents alanine mutagenesis region. Uppercase sequence represents original, non-mutated sequence.

>CYB5_YNL111C_WT

ATGCCTAAAGTTTATTCATATCAAGAAGTTGCAGAGCATAACGGTCCAGAAAATTTCTGG
ATTATAATTGACGACAAAGTTTATGATGTCTCTCAATTTAAGGACGAGCATCCAGGAGGA
GATGAGATTATAATGGACCTGGGAGGTCAAGACGCGACCGAAAGCTTCGTAGATATTGG
TCATAGTGATGAAGCCTTGAGACTTTTGAAGGGTTTGTACATAGGAGACGTCGATAAAAC
ATCCGAGAGGGTTTCAGTTGAGAAGGTCTCTACTTCCGAAAACCAATCCAAGGGCTCAG
GTACTTTAGTTGTTATTCTAGCAATCTTGATGTTGGGCGTGGCATATTATCTTTTGAACGA
ATAG

>CYB5_AM1_3K-8Q_9-24

ATGCCAgccgcagcagccgctgcaGAAGTTGCCGAACATAATGGTCCTGAAAACTTTTGGATTATC
ATCGATGACAAAGTATATGATGTGAGTCAATTTAAGGATGAACACCCTGGAGGTGACGA
GATTATCATGGACTTGGGTGGTCAAGACGCTACCGAGTCATTTGTAGATATCGGTCATTC
AGACGAAGCTCTTAGATTATTAAAAGGATTGTATATTGGTGACGTAGACAAAACCTCTGA
AAGAGTATCTGTAGAGAAGGTTAGCACAAGCGAAAATCAGTCAAAAGGAAGCGGCACT
CTAGTTGTTATCCTAGCTATATTGATGCTTGGTGTCGCATATTACTTGTTGAATGAATAG

>CYB5_AM2_12E-17E_36-51

ATGCCAAAAGTATATTCATACCAGGAGGTGGCTgctgctgcagcagcagcaAACTTTTGGATCATT
ATTGATGACAAAGTTTATGACGTCAGTCAATTCAAAGATGAACATCCCGGTGGGGACGA
AATTATAATGGATTTGGGCGGTCAAGACGCCACGGAGAGCTTCGTTGATATCGGACATTC
AGATGAGGCTTTGCGTTTACTAAAGGGACTTTATATTGGCGATGTTGACAAGACATCCGA
GCGTGTCTCTGTTGAGAAGGTGTCTACATCTGAAAATCAGTCTAAAGGCTCTGGTACACT
TGTGGTGATCTTGGCAATTCTGATGTTAGGTGTTGCTTATTACCTTTTAAATGAGTAA

>CYB5_AM3_21I-26K_63-78

ATGCCGAAGGTATATTCTTACCAGGAAGTTGCAGAACACAACGGACCAGAGAATTTCTG
GgcagcggctgctgctgctGTTTATGACGTCTCTCAATTTAAGGACGAACATCCTGGCGGTGACGA
AATTATAATGGATCTGGGGGGTCAAGATGCCACAGAATCTTTTGTGGATATCGGGCATAG
TGACGAAGCGTTGCGTCTACTGAAGGGCTTGTATATTGGCGATGTAGACAAAACTTCCGA
AAGAGTATCAGTGGAAAAAGTGAGTACATCTGAAAACCAAAGTAAGGGAAGTGGTACTT
TAGTTGTAATATTGGCCATTTTGATGCTTGGTGTAGCCTATTACCTACTGAACGAGTAA

>CYB5_AM4_30V-35D_90-105

ATGCCCAAGGTATATTCATACCAGGAAGTGGCGGAACACAATGGTCCAGAAAATTTTTG
GATTATCATAGATGACAAAGTTTACGATgctgctgccgcagcagcaGAGCACCCAGGTGGTGATGA
AATTATTATGGATTTGGGGGGCCAAGATGCCACTGAGAGCTTCGTCGATATAGGTCATTC
CGATGAAGCCCTGAGATTATTAAAGGGGTTGTATATTGGCGATGTTGACAAAACTTCTGA
GAGAGTCTCCGTCGAGAAAGTTAGCACATCAGAAAACCAATCTAAAGGTAGTGGTACTC
TGGTTGTGATTTTGGCTATATTAATGCTAGGTGTCGCGTATTACTTGCTTAACGAATAA

>CYB5_AM5_39G-44I_117-132

ATGCCAAAAGTTTATTCATATCAAGAAGTCGCCGAACATAATGGTCCTGAAAATTTTTGG
ATAATTATAGATGATAAGGTGTATGATGTATCACAGTTCAAGGATGAGCATCCTgcagcagct

gcggcagctATGGATCTAGGTGGCCAAGATGCCACAGAAAGCTTTGTCGATATTGGCCACTCT
GACGAAGCGTTGAGATTATTGAAAGGTCTATACATCGGCGACGTCGATAAAACATCTGA
AAGAGTTAGTGTGGAAAAGGTGTCAACCTCTGAAAACCAAAGTAAAGGCTCTGGAACAT
TAGTGGTGATTCTGGCGATTTTGATGTTAGGAGTGGCTTACTATCTGCTTAATGAATGA

>CYB5_AM6_48G-53T_144-159

ATGCCTAAAGTCTATAGTTATCAAGAAGTTGCCGAACATAATGGTCCAGAGAACTTCTGG
ATAATAATAGATGATAAAGTGTATGACGTTTCCCAATTTAAAGATGAACATCCAGGTGGT
GATGAAATTATAATGGACTTAgccgctgctgctgcagcgGAATCTTTTGTTGATATTGGGCACTCCG
ACGAAGCTCTACGTCTTCTTAAAGGATTGTATATCGGAGATGTAGATAAGACAAGTGAA
AGAGTTTCTGTAGAGAAAGTTTCTACCTCTGAGAACCAGTCCAAGGGCTCAGGTACGTTG
GTAGTTATCCTGGCTATCCTTATGCTGGGGGTTGCCTATTACTTGTTAAACGAATGA

>CYB5_AM7_57V-62S_171-186

ATGCCAAAAGTCTATTCATATCAAGAGGTTGCTGAACATAACGGCCCAGAAAATTTCTGG
ATTATTATAGATGACAAGGTCTATGATGTGTCTCAATTTAAGGATGAACATCCCGGCGGG
GACGAGATTATTATGGATCTTGGGGGACAAGATGCAACCGAATCATTcgctgctgcggccgcagct
GATGAGGCTTTACGTCTGTTAAAGGGATTATACATCGGCGATGTTGATAAAACAAGCGA
GAGAGTCAGCGTAGAGAAAGTGAGCACTAGTGAAAATCAATCTAAGGGATCCGGTACAC
TTGTTGTAATTTTAGCTATTCTGATGTTAGGTGTAGCATACTACCTTCTGAATGAATAA

>CYB5_AM8_66L-71G_198-213

ATGCCGAAAGTTTATTCTTATCAGGAAGTCGCGGAGCATAACGGACCGGAGAATTTCTG
GATAATTATTGATGATAAAGTTTATGACGTTTCACAATTTAAAGACGAGCATCCAGGCGG
CGATGAAATAATTATGGATTTAGGCGGTCAGGATGCAACAGAGAGTTTTGTCGACATAG
GACATTCAGACGAGGCAgccgctgccgccgcagcaTTGTATATAGGGGATGTGGATAAAACTAGT
GAGCGTGTAAGCGTTGAAAAAGTTAGTACTTCAGAAAATCAGAGCAAGGGTTCAGGTAC
CTTGGTAGTTATCTTAGCTATCTTAATGTTAGGAGTAGCATACTATCTTCTTAATGAATAG

>CYB5_AM9_75G-80T_225-240

ATGCCGAAAGTCTACAGCTATCAAGAAGTTGCGGAACATAACGGCCCGGAAAACTTTTG
GATTATTATTGACGATAAGGTATATGATGTTTCTCAGTTTAAAGATGAACACCCAGGAGG
CGACGAAATAATTATGGACCTAGGCGGTCAAGATGCAACTGAAAGTTTTGTTGATATAG
GTCATAGCGATGAAGCGCTTAGGTTATTGAAGGGTCTATACATTgccgcagctgctgctgctAGTG
AGAGGGTCTCAGTTGAAAAAGTAAGTACATCCGAAAACCAATCCAAGGGTTCTGGGACC
TTGGTGGTTATCTTAGCAATCCTAATGCTTGGTGTTGCATATTACTTGTTGAATGAATAG

>CYB5_AM10_84V-89V_252-267

ATGCCTAAAGTATATTCTTATCAAGAAGTCGCCGAACATAACGGTCCAGAAAATTTTTGG
ATCATTATTGACGATAAGGTATATGATGTCTCCCAATTTAAAGATGAACATCCTGGAGGC
GATGAAATTATTATGGATTTAGGTGGACAAGACGCGACCGAATCATTTGTTGATATTGGC
CACTCTGATGAAGCTTTGAGGTTGTTAAAGGGATTGTACATAGGCGATGTCGATAAGACT
AGTGAAAGGgcggctgcagcagctgctAGCACCAGCGAGAATCAATCAAAAGGGAGTGGGACGCT
TGTTGTTATATTGGCTATTTTGATGCTAGGCGTTGCGTATTACCTATTGAACGAATGA

>CYB5_AM11_93E-97K_279-291

ATGCCAAAGGTTTATTCATACCAGGAAGTAGCGGAACACAATGGACCAGAAAATTTTTG
GATTATCATAGATGATAAAGTGTACGATGTTAGCCAATTTAAAGATGAACATCCCGGCG
GTGATGAGATCATCATGGACCTGGGAGGGCAAGACGCTACCGAGTCCTTTGTCGACATC
GGTCATAGCGATGAGGCACTGAGACTACTGAAGGGACTTTATATTGGAGACGTGGATAA
GACAAGTGAAAGAGTCTCAGTAGAGAAAGTTTCAACCAGCgcagccgcggccgctgccTCAGGTA
CTTTAGTGGTGATTCTAGCAATTTTGATGCTAGGTGTGGCATATTATCTTTTGAATGAGTA
A
