# Supplementary figures and images for: Identification and characterization of protein interactions with the major Niemann–Pick type C disease protein in yeast reveals pathways of therapeutic potential
Source: Genetics. 2023 Jul 13;225(1):iyad129. doi: 10.1093/genetics/iyad129 (PMC10471228; doi:10.1093/genetics/iyad129)

A.

|           | Ncr1-Cub-TF                                                                       |                                                                                   |                                                                                    |                                                                                     |                                                                                     |                                                                                     |                                                                                     |                                                                                     |                                                                                     |
|-----------|-----------------------------------------------------------------------------------|-----------------------------------------------------------------------------------|------------------------------------------------------------------------------------|-------------------------------------------------------------------------------------|-------------------------------------------------------------------------------------|-------------------------------------------------------------------------------------|-------------------------------------------------------------------------------------|-------------------------------------------------------------------------------------|-------------------------------------------------------------------------------------|
|           | Control                                                                           |                                                                                   |                                                                                    | Low                                                                                 |                                                                                     |                                                                                     | High                                                                                |                                                                                     |                                                                                     |
| NubI-Ost1 | 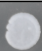 | 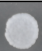 | 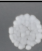 | 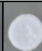 | 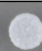 | 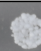 | 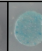 | 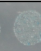 | 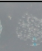 |
| NubG-Ost1 | 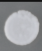 | 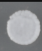 | 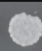 | 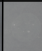 |                                                                                     |                                                                                     | 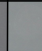 |                                                                                     |                                                                                     |

B.

Ncr1-Cub-YFP-TF

DIC

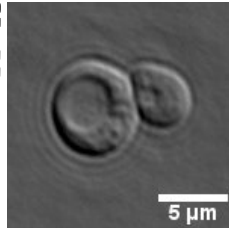

YFP

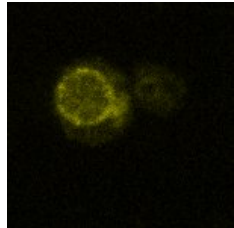

Supplement: iyad129_Supplementary_Data [file iyad129_supplementary_data.zip › Figure_S2_GENETICS-2023-306236.pdf]

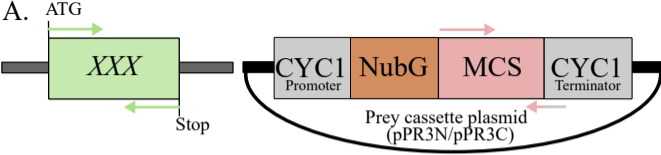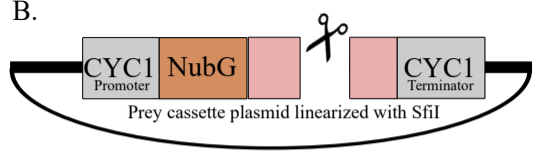

C. Co-transformation in yeast for prey construction by homologous recombination

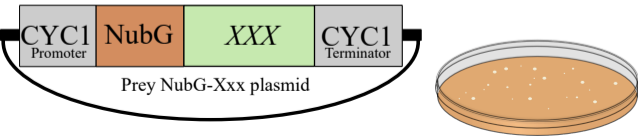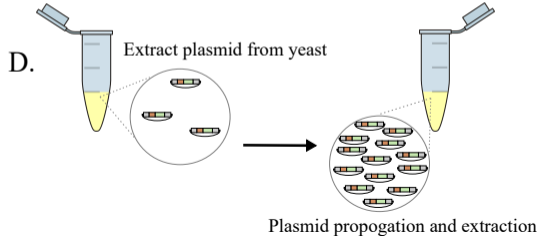

Supplement: iyad129_Supplementary_Data [file iyad129_supplementary_data.zip › Figure_S3_GENETICS-2023-306236.pdf]
